# Neurons and glial cells acquire a senescent signature after repeated mild traumatic brain injury in a sex-dependent manner

**DOI:** 10.3389/fnins.2022.1027116

**Published:** 2022-11-03

**Authors:** Nicole Schwab, Daria Taskina, Emily Leung, Brendan T. Innes, Gary D. Bader, Lili-Naz Hazrati

**Affiliations:** ^1^Department of Laboratory Medicine and Pathobiology, University of Toronto, Toronto, ON, Canada; ^2^Program in Neurosciences and Mental Health, The Hospital for Sick Children, Toronto, ON, Canada; ^3^The Donnelly Centre, University of Toronto, Toronto, ON, Canada; ^4^Department of Molecular Genetics, University of Toronto, Toronto, ON, Canada

**Keywords:** mild traumatic brain injury, cellular senescence, senolytic, concussion, sex difference

## Abstract

Mild traumatic brain injury (mTBI) is an important public health issue, as it can lead to long-term neurological symptoms and risk of neurodegenerative disease. The pathophysiological mechanisms driving this remain unclear, and currently there are no effective therapies for mTBI. In this study on repeated mTBI (rmTBI), we have induced three mild closed-skull injuries or sham procedures, separated by 24 h, in C57BL/6 mice. We show that rmTBI mice have prolonged righting reflexes and astrogliosis, with neurological impairment in the Morris water maze (MWM) and the light dark test. Cortical and hippocampal tissue analysis revealed DNA damage in the form of double-strand breaks, oxidative damage, and R-loops, markers of cellular senescence including p16 and p21, and signaling mediated by the cGAS-STING pathway. This study identified novel sex differences after rmTBI in mice. Although these markers were all increased by rmTBI in both sexes, females had higher levels of DNA damage, lower levels of the senescence protein p16, and lower levels of cGAS-STING signaling proteins compared to their male counterparts. Single-cell RNA sequencing of the male rmTBI mouse brain revealed activation of the DNA damage response, evidence of cellular senescence, and pro-inflammatory markers reminiscent of the senescence-associated secretory phenotype (SASP) in neurons and glial cells. Cell-type specific changes were also present with evidence of brain immune activation, neurotransmission alterations in both excitatory and inhibitory neurons, and vascular dysfunction. Treatment of injured mice with the senolytic drug ABT263 significantly reduced markers of senescence only in males, but was not therapeutic in females. The reduction of senescence by ABT263 in male mice was accompanied by significantly improved performance in the MWM. This study provides compelling evidence that senescence contributes to brain dysfunction after rmTBI, but may do so in a sex-dependent manner.

## Introduction

Mild traumatic brain injury (mTBI) is associated with short-term and long-term cognitive dysfunction with increased risk of neurodegenerative disease, especially when experienced repeatedly (Gupta and Sen, 2016). The pathophysiological mechanisms driving long-term cognitive dysfunction and neurodegeneration after mTBI are unclear, though pathological changes in the immediate period following injury have been elucidated. Indeed, what has been termed the secondary injury cascade after mTBI involves axonal damage, microglial activation, cell death, oxidative stress, and neuroinflammation (Pearn et al., 2017; Stojanovski et al., 2019b; Velayudhan et al., 2021; George et al., 2022). More recently, cellular senescence has been identified as a feature of this secondary injury cascade (Ritzel et al., 2019; Schwab et al., 2019, 2021; Tominaga et al., 2019).

Cellular senescence is defined as a state of cell cycle arrest accompanied by morphological changes, nuclear disorganization, metabolic alterations reflecting increased glycolysis, ROS generation, protein synthesis, mitochondrial dysfunction, and the secretion of pro-inflammatory factors collectively called the senescence-associated secretory phenotype (SASP) (Childs et al., 2017). Senescence can be induced by persistent DNA damage signaling through the DNA damage response (DDR), which activates cell cycle control proteins p53, p21, and p16 and their downstream effectors (Kumari and Jat, 2021). Senescent cells are characterized by anti-apoptotic features known as senescent cell anti-apoptotic pathways (SCAPs), that prevent them from apoptosis despite the accumulation of genomic and mitochondrial DNA damage (Zhu et al., 2015). Known and targetable SCAPs include the BCL-2 family of apoptotic proteins, PI3K/Akt, p53/p21/Serpine, HIF1α, and HSP90 (Kirkland et al., 2017). These pathways are often targeted in therapies aimed at reducing or eliminating senescence, as this would lead to apoptosis of SCAP-expressing cells (Kirkland and Tchkonia, 2020). Senescent cells have been shown to accumulate in the brains of patients with Alzheimer’s disease (AD) (Guerrero et al., 2021), Parkinson’s disease (PD) (Chinta et al., 2018), amyotrophic lateral sclerosis (ALS) (Vazquez-Villaseñor et al., 2020), mood disorders (Diniz et al., 2019), stroke (Torres-Querol et al., 2021), and traumatic brain injury (Ritzel et al., 2019; Schwab et al., 2019. Tominaga et al., 2019; Schwab et al., 2021).

There is emerging evidence that cellular senescence is involved in the pathogenesis of mTBI. This was first published by our lab in a cohort of post-mortem male athletes with a history of mTBI (Schwab et al., 2019) and has since been shown in mouse models by us (Schwab et al., 2021) and others (Ritzel et al., 2019; Tominaga et al., 2019). Although DNA damage-induced cellular senescence has clearly been identified as a mechanism driving brain dysfunction after mTBI, uncertainties remain. Indeed, which cell types become senescent and their respective functional repercussions remain unclear. Furthermore, senolytic drugs that target SCAP-expressing senescent cells for apoptosis require further investigation as they represent a potential treatment for individuals who remain symptomatic post-mTBI and at risk of developing a neurodegenerative disease. In recent years, senolytic drugs have been shown to reduce pathology and improve cognitive abilities in a mouse model of AD (Zhang et al., 2019), improve lifespan and measures of frailty in aged mice (Xu et al., 2018), and reduce infarct volume and improve cognitive outcomes in a mouse model of stroke (Lim et al., 2021), suggesting this treatment may also be beneficial in mTBI. To address these gaps, this manuscript uses a mouse model of repeated mTBI (rmTBI) to explore cellular senescence in the injured brain. Using various molecular techniques and single cell RNA sequencing (scRNAseq) we show that the DNA damage response and cellular senescence are early events following rmTBI, in both glial and neuronal cell types. We have also shown that administering the senolytic drug ABT263 1 week following injury improves neurobehavioral function and reduces markers of the DDR and senescence. This manuscript provides compelling evidence of a causal link between cellular senescence and neurobehavioral dysfunction after rmTBI, and highlights novel sex differences which may explain underlying sex differences in mTBI outcomes and highlight potential personalized therapeutic strategies.

## Materials and methods

### Animals

All experiments were approved by the Centre for Phenogenomics Animal Care Committee. Adult (8–10 week old) female and male C57BL/6 mice, housed randomly in the same room, were used in this study. Mice were kept under standard laboratory conditions with access to food and water *ad libitum* and a 12 h light/dark schedule (all surgical and behavioral work was performed during the light cycle). Separate groups of mice were used for molecular analysis (*n* = 4 per group unless otherwise stated) and behavior (*n* = 8 per group, unless otherwise stated) to avoid potential confounding effects of behavioral testing on the brain.

### Repeated mild traumatic brain injury model

Mice were randomly assigned to receive rmTBI with a closed-skull controlled cortical impact model or sham procedures. All mice received pre-operative subcutaneous injections of sustained release buprenorphine (1.2 mg/kg) then anesthetized with isoflurane (induced at 4% and maintained at 2%). Once anesthetized, mice were injected subcutaneously with lactated ringers containing 5% dextrose (0.75 ml) and under the scalp with equal parts xylocaine and bupivacaine (total injection volume of 0.1 ml). Mice were placed in a stereotactic frame (Stoelting) with a warmer ready base to maintain animal body temperature, preventing movement of the head with non-rupture ear bars. Once secured in place, a midline incision was made to expose the skull. Once the skull was leveled to the horizontal plane the desired location (2.5 mm right of Bregma, over the right somatosensory cortex) was identified and an impact of 2 m/s was administered with an electromagnetically driven Impact One Stereotaxic Impactor (Leica, Buffalo Grove, IL) using a 5 mm metal tip, to a depth of 1.2 mm, and with a dwell time of 200 ms. After suturing the midline incision closed, mice were placed in a recovery cage on their back, and righting reflex (total time taken for the mouse to flip over onto all four limbs) was recorded. Sham surgeries underwent the same procedure (including injections, incision, and isoflurane exposure), omitting the actual impact to the head. This procedure was repeated three times, 24 h apart, to give a total of three impacts or equivalent sham surgeries to the mice.

### Neurobehavioral testing

One week following the final injury, mice were subjected to neurobehavioral testing first with the light dark test (day 1 of testing) and the Morris Water Maze (MWM) test (days 2–10 of testing). Each testing day began with cages placed inside of the testing room for at least 30 min prior to testing, to acclimate the mice to the testing environment. The light dark test was used to assess exploratory behaviors and anxiety-like behaviors. For this test an arena (43.5 cm^2^) was prepared such that half of the space was bright (200 LUX) and half of the arena was darkened by a black box. Mice were placed in the arena for 10 min and their activity was monitored by infrared tracking. Time spent in each of the components (light or dark) was measured. The next day, MWM test was prepared to evaluate the mice for spatial memory and learning. The maze was positioned inside a pool centrally under the tracking device and approximately 1 meter away from surrounding walls with four different visual cues. The pool was filled with water to approximately 10 cm from the edge (providing mice with a clear view of external cues), heated to 25°C, and colored with non-toxic white paint to make the platform, placed in the centre of a target quadrant, invisible. The MWM test began on testing day 2 with a familiarization day, in which the platform remained visible above the water. The familiarization procedure entailed the mice standing on the platform for 60 s, followed by a single session containing four trials from different starting positions (pseudo-randomly predetermined N, S, E, W), followed again by a 60 s period on the platform. On days 3–5 mice underwent a training phase for the learning paradigm. In this time, each mouse underwent four blocks of three trials (a total of 12 trials per day per mouse) with approximately 15 min of rest between trial blocks. Time taken to reach the hidden platform (escape latency) was measured and swim speed was assessed. The learning probe day was on day 6, where the platform was removed and the number of goal crossings and distance to the goal was measured. Days 7–9 were again training days but this time for the reversal learning paradigm, in which the platform location was changed and the time taken to reach the new platform location was measured. The final testing day was the reversal learning probe day, in which the platform was removed, and the number of goal crossings and distance to the goal was again measured.

### Senolytic intervention

A group of male rmTBI and sham mice received a single intraperitoneal injection at 1-week post-injury containing either the senolytic agent ABT263 (1.5 mg/kg, dissolved in PEG-400, ddH_2_O, and DMSO) or a vehicle. The rationale for a single dose of the drug ABT263 was a recent publication indicating that a single systemic dose of ABT263 is sufficient to eliminate senescent cells in the brain and improve behavior (Fatt et al., 2022). One week after injection, a total of 2 weeks post-injury, mice underwent the MWM paradigm, as described above, or molecular analysis, as described below.

### Animal sacrifice

Following the three impacts or sham surgeries, mice were sacrificed for tissue analysis at 1 week after the final injury, and mice used for behavioral testing (not included in tissue analyses) were sacrificed on the final day of behavioral testing. For molecular analysis, mice were sacrificed via transcardial perfusion with PBS and heparin under ketamine (150 mg/kg) and xylazine (10 mg/kg). Following perfusion, the brain was immediately removed, cut into two hemispheres, and flash frozen in liquid nitrogen in a bath of isopentane and stored at –80°C. For histological analysis, mice underwent the same perfusion protocol followed by an additional perfusion with 4% paraformaldehyde, followed by 24H post-fixation in 4% PFA. These fixed brains were then cut into two hemispheres and processed in paraffin embedded blocks.

### Histology

Formalin-fixed paraffin embedded blocks from the ipsilateral side of each mouse brain were used for immunohistochemistry. Sagittal blocks were cut into six-micron sections and mounted on glass slides. Sections were stained with hematoxylin and eosin (H&E), glial fibrillary acidic protein (GFAP, Dako kit, KAC0002), ionized calcium binding adaptor molecule 1 (IBA1, FujiFilm Wako #019-19741 1:5000), and γH2AX (Millipore, 1:1,000, JBW301). Quantification of histology images was performed using QuPath software by taking three ROIs from the cortical region near impact, estimating stain vectors using automated pre-processing, followed by an automated positive pixel count analysis. Differences in staining were determined by percent of positive pixels detected.

### Western blot and dot blot

For Western Blot (WB) the ipsilateral cortex and underlying hippocampus near the impact region was homogenized and sonicated in ice-cold PBS containing a protease and phosphatase inhibitor cocktail (Thermofisher). The supernatants were harvested after centrifugating at 12,000 *g* for 20 min. Protein concentration was measured using a protein assay kit (Biorad). Protein samples were loaded in 10% polyacrylamide gels (Biorad) and transferred to a nitrocellulose membrane (Biorad). Membranes were blocked in 2% BSA of tris buffered saline with 0.05% tween-20 (TBS-T) for 2 h and incubated with primary antibodies for 2 h at room temperature or overnight at 4°C. HRP conjugated secondary antibodies [Sigma, anti-mouse (A9044, 1:10,000) and rabbit (A0545, 1:20,000)] were employed. Membranes were visualized by the Odyssey Fc imaging system (LI-COR). Primary antibodies were 53BP1 (Novus biologicals, NB100-304, 1:10,000), DNA2 (Thermofisher, PA5-68167, 1:2,000), p21 (BD biosciences, 556430, 1:1,000), p16 (Thermofisher, PIMA517142, 1:1,000), γH2AX (Cell signaling technology, 2577S, 1:1,000), HMGB1 (Abcam, ab18256, 1:1,000), Mouse-Reactive STING Pathway Antibody Sampler Kit (Cell signaling technology, 16029T), and GAPDH (Cell signaling technology, 2118S, 1:1,000). Targets were normalized to GAPDH to control for loading.

For the Dot Blot, the same brain region as used in Western Blot analysis was used and dsDNA was isolated by first isolating the nuclear fraction followed by purification of genomic DNA containing DNA-RNA hybrids. dsDNA was measured by NanoDrop One (Thermofisher) and diluted in elution buffer for final concentrations of 200, 100, 50, 25, and 12.5 ng. Three nitrocellulose membranes were spotted with 2 uL of each sample dilution and dried for 1 h, followed by 2 h incubation in 1% BSA in TBST, then incubation in primary antibodies in 1% BSA in TBST overnight at 4°C. The primary antibodies were as follows: anti-dsDNA for detection of DNA (1:20,000 dilution, Abcam AB27156), anti-S9.6 for detection of R-Loops (1:5,000, Sigma MABE1095), and anti-DNA/RNA oxidative damage (1:4,000, Abcam ab62623). Membranes were incubated in HRP-conjugated secondaries (anti-mouse, 1:10,000 dilution, source) for 2 h followed by a 15 min wash with PBST and two 15 min PBS washes. Finally, membranes were developed with enhanced chemiluminescence (ECL) substrate (Bio-Rad) and blots were captured with the Odyssey Fc imaging system (LI-COR). Targets were normalized to dsDNA to control for loading.

### Single cell ribonucleic acid sequencing

The ipsilateral hemisphere, excluding brainstem and cerebellum, were incubated in lysis buffer before homogenization. Isolated nuclei were washed twice with cold wash and resuspension buffer and then filtered through a 40 um filter. Nuclei were stained with DAPI and flow sorted. Sorted nuclei were used as input into the 10X Genomics single-cell 3′ v3.1 assay and processed as outlined by user guide. Briefly, following counting, the appropriate volume for each sample was calculated for a target capture of 10,000 nuclei. After droplet generation, samples were transferred onto a pre-chilled 96 well plate (Eppendorf), heat sealed and incubated overnight in a Veriti 96-well thermos cycler (Thermo Fisher). The next day, sample cDNA was recovered using Recovery Agent provided by 10x and subsequently cleaned up using a Silane DynaBead (Thermo Fisher) mix as outlined by the user guide. Purified cDNA was amplified for 11 cycles before being cleaned up using SPRIselect beads (Beckman). Samples were diluted 4:1 [elution buffer (Qiagen):cDNA] and run on a Bioanalyzer (Agilent Technologies) to determine cDNA concentration. cDNA libraries were prepared as outlined by the Single Cell 3′ Reagent Kits v3.1 user guide with modifications to the PCR cycles based on the calculated cDNA concentration. The molarity of each library was calculated based on library size as measured bioanalyzer (Agilent Technologies) and quantitative polymerase chain reaction (qPCR) amplification data (Roche). Samples were pooled and normalized to 1.5 nM. Library pool was denatured using 0.2N NaOH (Sigma) for 8 min at room temperature, neutralized with 400 mM Tris-HCL (Sigma). Library pool at a final concentration of 300 pM were loaded to sequence on Novaseq 6000 (Illumina). Samples were sequenced with the following run parameters: Read 1–28 cycles, Read 2–90, index 1–8 cycles.

After deconvolution and quantification using the 10X Genomics CellRanger software, the data were filtered to include only cells with between 500 and 2,500 genes detected, with less than 5% of transcripts contributed by mitochondrial genes. All samples were normalized together using the clustered pool and deconvolute approach available in the scran R package (Lun et al., 2016). Highly variable genes were identified by their deviation from the fitted mean-variance relationship. Principal component analysis was used to further summarize each cell’s transcriptome, and both 2D embeddings and clustering was performed from these principal components as implemented in the Seurat R package (Hao et al., 2021). Clustering parameter optimization was performed as outlined in scClustViz (Innes and Bader, 2018). Differential expression between rmTBI treatment and sham was performed using MAST (Finak et al., 2015) to fit a hurdle model with a random effect term for individuals to effectively address pseudoreplication bias (Zimmerman et al., 2021). Cell types were annotated based on a published mouse brain atlas (Zeisel et al., 2018) using scpred (Alquicira-Hernandez et al., 2019). Gene Set Enrichment Analysis (Subramanian et al., 2005) was performed using a custom gene set collection designed for Enrichment Map (Merico et al., 2010) containing both GO biological process terms as well as a compilation of pathway databases available here: http://download.baderlab.org/EM_Genesets/January_01_2020/Mouse/symbol/Mouse_GOBP_AllPathways_no_GO_iea_January_01_2020_symbol.gmt. This gene set was supplemented by custom gene sets defining senescence core and effector genes, as well as genes associated with the SASP (Coppé et al., 2010). Results from differential gene expression and gene set enrichment analysis can be found in Supplementary files 1 and 2, respectively.

### Statistical analysis

Righting reflex was assessed with a two-way ANOVA using injury and sex as variables. Differences between GFAP and Iba-1 staining were assessed with unpaired *t*-tests. For the light dark test, raw data from Ethovision (Noldus) was obtained and the total time spent in the dark chamber was statistically compared using a two-way ANOVA with injury and sex as variables. For the MWM, raw data from Ethovision (Noldus) was obtained and analyzed in R, using the RTrack package (Overall et al., 2020). The package utilizes a machine learning algorithm to classify swim paths into strategies including goal-oriented, procedural, and non-goal-oriented types. Differences in escape latency were statistically assessed with three-way ANOVAs using injury, sex, and training day as variables and probe trials were assessed with two-way ANOVAs using injury and sex as variables. Differences in search strategy uses were assessed by performing a Chi-Square analysis on the total number of search strategy uses over the entire training period. Western blot data was assessed by normalizing targets to GAPDH followed by a two-way ANOVA with injury and sex as variables. Statistical analysis of scRNAseq data is described in the previous subsection.

## Results

### Repeated mild traumatic brain injury is induced by closed skull impact model in mice

**FIGURE 1:**
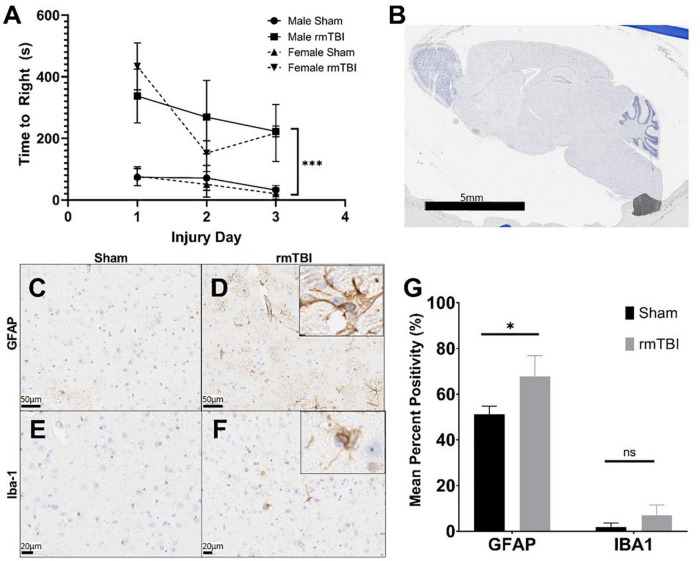
Injury induced by closed skull impact model is mild. The injury induced by the presented model can be deemed mild, as indicated by righting reflex measured immediately after injury. rmTBI mice took significantly longer to right than shams **(A)** on each day with sex having no effect (*p* < 0.001, main effect of injury and main effect of day, three-way ANOVA, *n* = 6 per group). Also supportive of a mild injury, despite the absence of any gross lesion **(B)**, rmTBI mice showed significantly increased GFAP staining (*p* = 0.04, unpaired *t*-test, *n* = 4 per group) **(C,D,G)**, and an increase in lba-1 staining that did not reach statistical significance (*n* = 4 per group) **(E–G)** in the cortical region near impact. Error bars represent standard deviation. Indication of statistical significance is as follows: *p < 0.05.

The injuries sustained by mice in this study were deemed mild, as injury led to attenuated righting reflex under 15 min (Namjoshi et al., 2014). Injured mice took significantly longer to right than shams on each of the three injury days (*p* < 0.001, main effect of injury, two-way ANOVA) (Figure 1A). Over the three injury days, injured mice took on average 267.9 s to right compared to only 54.0 s in shams. There was no significant effect of sex on righting reflex by way of two-way ANOVA.

Despite the absence of a visible lesion (Figure 1B), injured mice showed other evidence of mild injury. rmTBI mice had increased GFAP staining in the cortex and hippocampus (Figures 1C,D), which was significantly elevated compared to shams in the cortical region (*p* = 0.04, unpaired *t*-test) (Figure 1G). In addition to significantly increased positivity, GFAP-positive astrocytes in injured mice frequently displayed ramification and branching (Figure 1D inset) typical of the astrogliosis response initiated by mTBI. Positive staining for Iba-1 in the same brain regions was increased in injured mice compared to shams (Figures 1E,F), although this did not reach statistical significance in the cortical region (Figure 1G). Some Iba-1-positive microglia in the injured brain displayed altered morphologies, including ramification and elongation (Figure 1F inset). These changes suggest the presence of a mild injury induced by this model.

### Cognitive dysfunction and behavioral disinhibition 1 week after rmTBI

**FIGURE 2:**
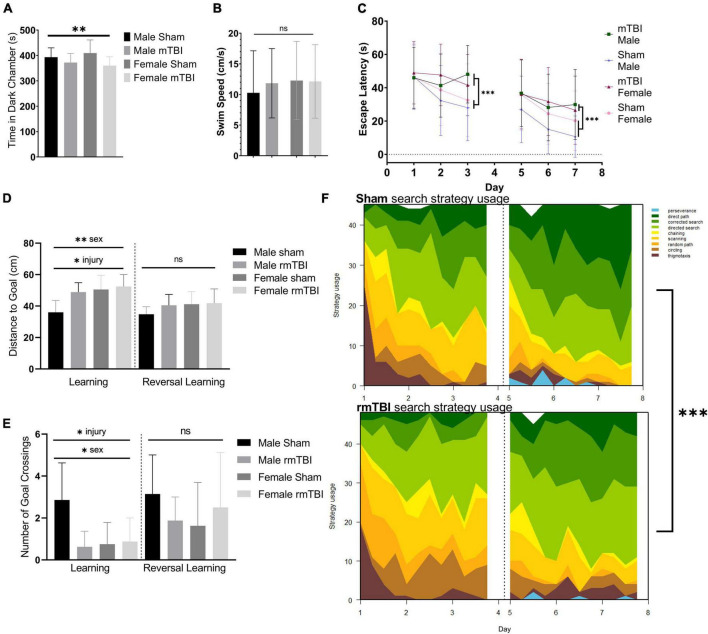
Sex-specific cognitive dysfunction and behavioral disinhibition 1 week after rmTBI. In the light-dark box test **(A)**, rmTBI mice spent significantly longer in the light chamber compared to shams (*p* = 0.04, main effect of injury, two-way ANOVA), with no main effect of sex. In the Morris Water Maze (MWM), no significant differences in swim speed were present between groups **(B)**. In the learning and reversal learning trials, rmTBI mice spent significantly longer to reach the platform compared to shams (*p* < 0.001, main effect of injury, three-way ANOVA) over trial days (*p* < 0.001, main effect of training day, three-way ANOVA) **(C)**. There was a small effect of sex nearing significance (*p* = 0.1, three-way ANOVA), and therefore a significant interaction between injury, sex, and training day (*p* = 0.05, three-way ANOVA) reflecting baseline differences between male and female sham mice. On the learning probe days, rmTBI mice had a significantly higher distance to the goal compared to shams (*p* < 0.01, main effect of injury, two-way ANOVA) **(D)**. This measurement also showed a sex difference (*p* < 0.01, main effect of sex, two-way ANOVA) and a significant interaction between sex and injury (*p* = 0.05, two-way ANOVA). Post-hoc testing indicates a significant difference between male shams vs male rmTBI mice (*p* = 0.01, Tukey HSD), male shams vs female shams (*p* = 0.005, Tukey HSD), and male shams vs female rmTBI mice (*p* = 0.001, Tukey HSD). Similarly, rmTBI mice had significantly less number of goal crossings compared to shams (*p* = 0.02, main effect of injury, two-way ANOVA) **(E)** with an effect of sex (*p* = 0.04, main effect of sex, two-way ANOVA) and a significant interaction between sex and injury (*p* = 0.01, two-way ANOVA). Similar to distance to goal, post-hot testing indicates a significant difference between male and female shams (*p* = 0.01, Tukey HSD), male shams and male rmTBI mice (*p* = 0.007, Tukey HSD), and male shams and female rmTBI mice (*p* = 0.02, Tukey HSD). Reversal probe tests were not significantly different between shams and rmTBI mice. Using a machine learning classifier (RTrack), search strategy was assessed over training days between sham and rmTBI mice, without consideration for sex **(F)**. Overall, rmTBI mice used more goal-oriented strategies (perserverence, direct path, corrected search, and directed search) and less procedural (chaining, scanning) and non-goal-oriented (random path, circling, thigmotaxis) strategies compared to sham mice in total (*p* < 0.0001, *x*^2^ = 39.95, Chi Square). Significant differences between rmTBI and sham mice were found on day 2 (*p* = 0.003, *x*^2^ = 11.83), day 3 (*p* < 0.0001, *x*^2^ = 22.51), day 6 (*p* = 0.02, *x*^2^ = 8.25), and day 7 (*p* = 0.004, *x*^2^ = 11.25). Error bars represent standard deviation with *n* = 8 per group. Indication of statistical significance is as follows: *p < 0.05, ^**^p < 0.01, ^***^p < 0.001. ns, not significant.

At 1-week post-injury, injured mice displayed behavioral disinhibition, defined as a tendency toward risk-taking, novelty-seeking, and negligence (Iacono et al., 2003) as measured by the light dark box task. In this test, injured mice spent significantly more time in the light chamber and less time in the dark chamber compared to shams (*p* = 0.04, two-way ANOVA) with no significant effect of sex (Figure 2A).

Injured mice displayed significant cognitive impairment in the MWM task 1 week following injury (Figures 2B–F). No significant differences in swim speed (velocity) were found between groups (two-way ANOVA; Figure 2B). Across the training days in the learning paradigm, there was a significant main effect of injury on escape latency (*p* < 0.001, three-way ANOVA) with injured mice taking significantly longer to find the hidden platform compared to shams (Figure 2C), as well as a main effect of training day (*p* < 0.001, three-way ANOVA). A small effect of sex was seen nearing significance (*p* < 0.1, three-way ANOVA) resulting in a significant interaction between injury, sex, and training day (*p* = 0.05, three-way ANOVA) reflecting baseline differences between male and female sham mice in which female sham mice are performing worse than their male counterparts. In the learning probe test, rmTBI mice had a significantly higher distance to the goal compared to sham mice (*p* < 0.01, main effect of injury, two-way ANOVA) (Figure 2D). This measurement also showed a sex difference (*p* < 0.01, main effect of sex, two-way ANOVA) and a significant interaction between sex and injury (*p* = 0.05, two-way ANOVA). *Post-hoc* testing indicates a significant difference between male shams and male rmTBI mice (*p* = 0.01, Tukey HSD), male shams and female shams (*p* = 0.005, Tukey HSD), and male shams vs female rmTBI mice (*p* = 0.001, Tukey HSD). Similarly, rmTBI mice had a significantly less number of goal crossings compared to shams (*p* = 0.02, main effect of injury, two-way ANOVA; Figure 2E) with a main effect of sex (*p* = 0.04, two-way ANOVA) and a significant interaction between sex and injury (*p* = 0.01, two-way ANOVA). Similar to distance to the goal, *post hoc* testing indicates a significant difference between male and female shams (*p* = 0.01, Tukey HSD), male shams and male rmTBI mice (*p* = 0.0007, Tukey HSD), and male shams and female rmTBI mice (*p* = 0.02, Tukey HSD). In the reversal probe test, there were no significant differences in mean distance to the goal nor number of goal crossings, although both male and female rmTBI mice had a slightly higher distance to the goal compared to shams (Figures 2D,E).

Search strategy analysis further confirmed the presence of cognitive and executive dysfunction in injured mice compared to shams (Figure 2F). Three main categories of search strategies were assessed. Non-goal-oriented strategies include thigmotaxis, circling, and random paths, indicating that the mouse is not attempting to reach the goal but is instead motivated to escape the pool. Procedural strategies, including scanning and chaining, indicating that the mouse is aware of a goal, but is using egocentric measures to find it. Last, allocentric strategies including direct searches, corrected searches, and direct paths indicate that the mouse is orienting itself based on cues around the room to find the goal. rmTBI mice used significantly more non-goal-oriented strategies and less goal-oriented strategies compared to shams overall (*p* < 0.0001, χ^2^ = 39.95, Chi-Square analysis) (Figure 2E). For the learning paradigm on the first day of training, use of search strategies was not significantly different between rmTBI mice and shams. However, rmTBI and sham mice utilized significantly different strategies on training days 2 (χ^2^ = 11.83, *p* = 0.003) and 3 (χ^2^ = 22.51, *p* < 0.001). From training day one to training day three sham mice increased the use of goal-oriented strategies by 32.0%, while rmTBI mice only increased the use of goal-oriented strategies by 17.7%. For the reversal learning paradigm, there was no significant difference between search strategy usage on day 5, but a significant difference on days 6 and 7 (χ^2^ = 8.25, *p* = 0.016 for day 6, χ^2^ = 11.25, *p* = 0.003 for day 7). From the first day of reversal learning training on day 5 to the final day of training on day 7, sham mice increased the use of goal-oriented strategies by 21.0%, whereas rmTBI mice only increased the use of these strategies by 17.0%. Together, these data indicate that deficits in both spatial memory and executive dysfunction occur 1 week following rmTBI.

### Multiple forms of deoxyribonucleic acid damage accumulate 1 week post-injury

**FIGURE 3:**
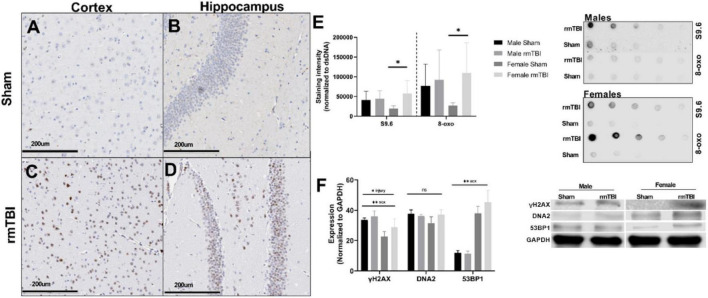
Deoxyribonucleic acid (DNA) damage accumulates near the region of impact 1 week after rmTBI. DNA damage is evident 1 week post-injuries. Immunohistochemistry for yH2AX in female mice reveals DNA damage in the form of DSBs accumulates after rmTBI in both the cortex at the impact region (**A,C**, scale bar represents 200 μm) and underlying hippocampal dentate gyrus (**B,D**, scale bar represents 200 μm), with minimal staining in shams. Staining of dsDNA with S9.6 to assess levels of R-Loop structures revealed a significant main effect of injury (*p* = 0.05, two-way ANOVA), and post-hoc testing indicates that this increased expression is only significant in the female rmTBI mice compared to their sham counterparts (*p* = 0.04, Bonferroni). Staining of dsDNA for 8-oxo to assess oxidative base damage shows a similar trend, with a main effect of injury nearing significance (*p* = 0.08, two-way ANOVA) and post-hoc testing revealing the increased expression to be significant only in female mice (*p* = 0.04, Bonferroni). Representative images of the dot blot are shown to the right of the quantification plot **(E)**. Using Western Blotting, protein levels of yH2AX were found to be significantly increased in rmTBI mice compared to controls **(F)** (*p* = 0.05, main effect of injury, two-way ANOVA), with a significant effect of sex (*p* = 0.0004, main effect of sex, two-way ANOVA) and no significant interaction. DNA2 trended toward increased expression in female rmTBI mice compared to shams, but did not reach statistical significance **(F)**. 53BP1 was found to have a significant main effect of sex (*p* < 0.0001, two-way ANOVA) but no significant main effect of injury, however, it was trending toward increased expression in female rmTBI mice compared to shams. Representative blots are shown to the right of the plot **(F)**. Error bars represent standard deviation and there are *n* = 4 per group. Indication of statistical significance is as follows: *p < 0.05.

Accumulation of DNA damage in the form of double-strand breaks (DSBs), oxidative lesions, and R-loops was apparent as early as 1-week post-injury. Immunohistochemistry for the γH2AX, a marker of DSBs, revealed extensive staining in the cortex and hippocampus underlying the impact region in rmTBI mice (Figures 3C,D) but not in shams (Figures 3A,B). Injured mice also showed significant increased levels of R-Loops (*p* = 0.05, two-way ANOVA; Figure 3E), three-stranded nucleic acid structures comprised of a DNA-RNA hybrid and a displaced DNA loop which are a key source of genomic instability and DNA damage (Hegazy et al., 2020). *Post hoc* testing revealed that this increase was only statistically significant in female mice (*p* = 0.04, Bonferroni; Figure 3E). Similarly, rmTBI mice showed evidence of increased oxidative DNA damage, marked by 8-hydroxy-2′-deoxyguanosine, that trended toward significance (*p* = 0.08) compared to shams (Figure 3E). Again, *post hoc* testing revealed a significant increase in 8-oxo in female rmTBI mice only (*p* = 0.04, Bonferroni; Figure 3E).

Using Western Blotting, γH2AX was found to be significantly increased in rmTBI mice compared to controls (*p* = 0.05, main effect of injury, two-way ANOVA; Figure 3F) with a significant effect of sex (*p* = 0.0004, main effect of sex, two-way ANOVA) and no significant interaction. DNA2 was not statistically different between groups but trended toward increased expression in female rmTBI mice compared to shams (Figure 3F). 53BP1 also had a main effect of sex (*p* < 0.0001, two-way ANOVA) but no significant effect of injury, again trending toward increased expression in rmTBI female mice compared to shams.

Together, these data indicate the presence of multiple forms of DNA damage at 1-week post-injury, including DSBs, oxidative damage, and the accumulation of R-Loops, as well as the activation of a DNA damage response in a possibly sex-dependent manner.

### Cellular senescence is evident 1 week post-injury

**FIGURE 4:**
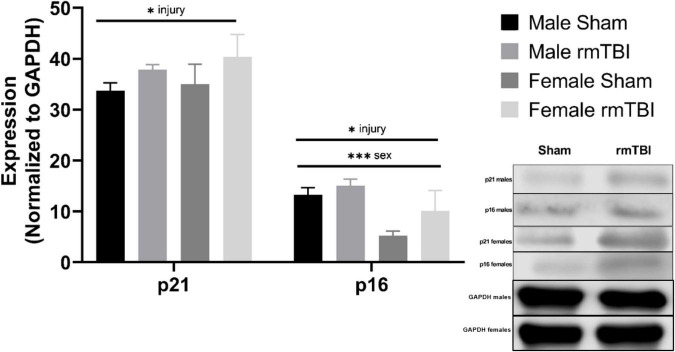
Cellular senescence is evident 1 week post-injuries. Using Western Blotting, protein levels of p21 and p16 were assessed. One week post-injuries p21 was significantly elevated in rmTBI mice compared to shams (*p* = 0.01, main effect of injury). There was no significant effect of sex on p21 expression. p16 expression was significantly increased in rmTBI mice compared to shams (*p* = 0.01, main effect of injury) and significantly differed between sexes (*p* = 0.001, main effect of sex) with no interactive effect. Post-hoc testing revealed significantly higher p16 expression in male shams compared to female shams (*p* = 0.001, Tukey HSD), female rmTBI compared to female shams (*p* = 0.05, Tukey HSD), and male rmTBI compared to female rmTBI (*p* = 0.04, Tukey HSD). All statistical values are results of two-way ANOVAs with *n* = 4 per sex-segregated group. Error bars represent standard deviation. Indication of statistical significance is as follows: ^#^*p* < 0.01, *p < 0.05, ^**^p < 0.01, ^***^p < 0.001.

The protein expression of cell cycle control and senescence regulator proteins p16 and p21 were assessed at 1-week post-injury as markers of cellular senescence (Figure 4). rmTBI mice showed significant protein elevation of p21 compared to shams (*p* = 0.01, main effect of injury, two-way ANOVA) with no effect of sex. Protein expression of p16 was also significantly increased by injury (*p* = 0.01, main effect of injury, two-way ANOVA) but had a main effect of sex as well (*p* = 0.001, main effect of sex, two-way ANOVA) with no interactive effect. *Post hoc* testing indicates significantly higher p16 expression in male shams compared to female shams (*p* = 0.001, Tukey HSD), female rmTBI compared to female shams (*p* = 0.05, Tukey HSD), and male rmTBI compared to female rmTBI (*p* = 0.04, Tukey HSD).

### cGAS-STING mediated type-1 interferon signaling 1 week post-injury

**FIGURE 5:**
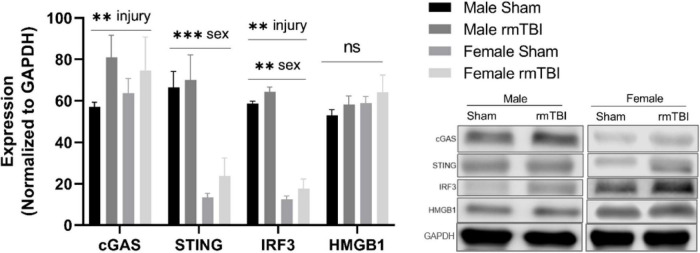
cGAS-STING mediated signaling 1 week post-rmTBI. Western blotting was used to quantify proteins involved in cGAS-STING signaling 1 week after rmTBI. cGAS was significantly increased in rmTBI mice compared to shams (*p* = 0.005, main effect of injury) with no sex differences. STING was significantly affected by sex (*p* < 0.0001, main effect of sex), and although trending toward increased expression did not reach statistical significance for effect of injury. Post-hoc analysis revealed STING was significantly increased in sham males compared to both sham and rmTBI females (both *p* < 0.0001, Tukey HSD), and rmTBI males compared to both sham and rmTBI females (both *p* < 0.0001, Tukey HSD). IRF3 was significantly increased by injury (*p* = 0.002, main effect of injury) and affected by sex (*p* < 0.0001, main effect of sex). Similar to STING, post-hoc analysis indicates that IRF3 expression was significantly higher in sham males compared to both sham females and rmTBI females (*p* < 0.0001, Tukey HSD). No statistical significant change for HMGB1 was observed. All statistical values are results of two-way ANOVAs with *n* = 4 per sex-segregated group. Error bars represent standard deviation and indication of statistical analysis is as follows: ^#^*p* < 0.1, *p < 0.05, ^**^p < 0.01, ^***^p < 0.001. ns, not significant.

We next assessed the expression of proteins associated with cGAS-STING signaling, a cytosolic DNA sensing pathway involved in initiating a type-I interferon response to genotoxic stress or viral infection which has been shown to be elevated in brain injury and neurodegenerative diseases (Barrett et al., 2021; Decout et al., 2021; Paul et al., 2021). One-week post-injury, cGAS protein level was significantly elevated in rmTBI mice compared to shams (*p* < 0.0001, main effect of injury, two-way ANOVA) with no sex differences (Figure 5). STING was not significantly altered by injury but instead was significantly higher in males compared to females (*p* < 0.0001, main effect of sex, two-way ANOVA; Figure 5). IRF3 was significantly increased in rmTBI mice compared to shams (*p* = 0.02, main effect of injury, two-way ANOVA) and was also higher in males compared to females (*p* < 0.0001, main effect of sex, two-way ANOVA; Figure 5). No statistically significant changes were found for HMGB1 (Figure 5).

### Single-cell ribonucleic acid sequencing of the injured mouse brain reveals senescent-like neurons

#### Identification of cell types and quality control

**FIGURE 6:**
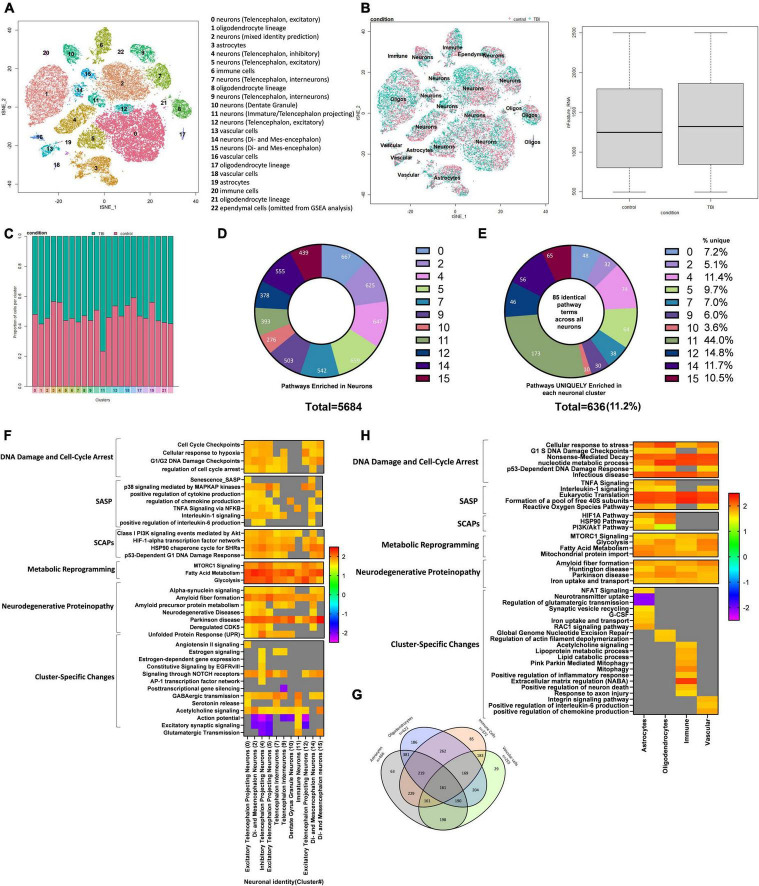
Single-Cell RNA sequencing of the injured mouse brain. A total of 23 unique cell clusters were identified **(A)**, with similar cellular composition and RNA feature counts between shams and rmTBI mice **(B,C)**. In total, 5,684 pathways were significantly enriched in neuronal cell types **(D)**, of which 636 (11.2%) were uniquely enriched in a single cluster **(E)**. Gene set enrichment analysis (GSEA) revealed a senescence-like expression profile in neuronal clusters **(F)** including DNA damage and cell cycle control, senescence-associated inflammation, senescence-associated anti-apoptotic mechanisms, and cell specific changes with potential functional repercussions. Glial cells shared a total of 161 enriched pathways alongside unique features for each cell type **(G)**. GSEA revealed a similar expression profile in glial cells to neurons, indicating senescence and cell-specific functional changes **(H)**.

To further characterize the pathophysiological mechanisms activated following rmTBI, we used unbiased high-throughput scRNAseq to compare the transcriptional profiles between rmTBI and sham mice 1-week post-injury. Data was obtained from the brain tissue of 2 rmTBI and 2 sham male mice. Cells with similar transcriptomes were grouped together using a recognized clustering algorithm and quality control was performed as described in the Methods section. Following quality control, 44830 cells (20959 from sham group, 23871 from rmTBI group) were grouped into 23 clusters with distinct profiles that mapped onto 6 major cell types: neurons, astrocytes, oligodendrocytes, immune cells, vascular cells, and ependymal cells (Figure 6A). Cell counts for each cell type between experimental groups are found in Table 1. A very small number of ependymal cells were identified, and so they were excluded from our gene set enrichment analysis (GSEA) analysis and interpretation due to lack of statistical power.

**TABLE 1:** Summary of cluster identities and cell count per cluster by condition.

Cluster	Identified cell type	Cell count (Total)	Cell count (Sham)	Cell count (mTBI)
0	Telencephalon projecting neurons	9,429	4,518	4,911
1	Oligodendrocytes	7,205	2,990	4,215
2	Di- and mesencephalon neurons	6,106	2,761	3,345
3	Astrocytes	3,409	1,929	1,480
4	Neurons – Telencephalon projecting neurons	2,786	1,562	1,224
5	Neurons – Telencephalon projecting neurons	2,300	1,006	1,294
6	Immune cells	2,215	1,002	1,213
7	Neurons – Telencephalon interneurons	1,929	827	1,102
8	Oligodendrocytes	1,651	775	876
9	Neurons – Telencephalon interneurons	1,363	597	766
10	Neurons – Telencephalon projecting neurons	1,287	654	633
11	Neurons – Immature neurons/telencephalon interneurons	895	209	686
12	Neurons – Telencephalon projecting neurons	861	395	466
13	Vascular cells	842	451	391
14	Neurons – Di- and Mesencephalon Neurons	721	337	384
15	Neurons – Di- and mesencephalon neurons	635	342	293
16	Vascular cells	388	229	159
17	Oligodendrocytes	216	101	115
18	Vascular cells	163	74	89
19	Astrocytes	125	70	55
20	Immune cells	119	52	67
21	Oligodendrocytes	101	43	58
22	Ependymal cells	84	35	49

#### Cellular composition is similar between rmTBI and sham animals

Cell identity was largely preserved in the injured brain, as indicated with unbiased clustering and a comparison of feature RNA count between rmTBI and sham mice (Figure 6B). Furthermore, the proportion of cells identified for each group per cluster confirmed a similar cellular composition between sham and injured brains (Figure 6C).

#### Identification of rmTBI-related pathways

We performed differential gene expression (DGE) analysis between sham and injured cells, then subsequently performed GSEA, a powerful and sensitive tool with higher sensitivity compared to the DGE analysis (Liu et al., 2018). GSEA aggregates information from broad sets of genes that are presumed to be functionally related. This analysis revealed several pathways which are significantly changed following brain injury.

Although cellular senescence is evident in the mouse brain 1-week post-injury, senescent cells likely only make up a small subset of cells in the injured brain, with their downstream repercussions having toxic effects to surrounding cells and slowly spreading the phenotype. As single-cell sequencing is inherently prone to incomplete detection of genes expressed at low levels, we do not expect senescence-related genes to be the top DEGs in this analysis. Therefore, to detect senescence, emphasis is made on pathways enriched in rmTBI using GSEA. Supplementary data files containing DEG analysis and GSEA are made available. Noteworthy, the number of cells in cluster 23 was insufficient for DEG and GSEA analysis, and so it was excluded from future results sections.

#### Senescent-like neurons emerge in the week following rmTBI

Whether or not neurons can become senescent is controversial and has been debated. Some studies have argued that neurons cannot truly become senescent due to their post-mitotic nature. To account for an ongoing debate on whether neurons can become senescent and/or take on a senescent-like phenotype (Fielder et al., 2017; Sah et al., 2021), we interpreted pathways enriched in glial cell and neuronal clusters separately and investigated differences in their senescence pathways. Neurons were classified by subtypes that were assigned by comparing transcriptomes to an existing mouse brain atlas (Zeisel et al., 2018). In total, 5684 pathways were enriched in neuronal cell types (Figure 6D). To first identify pervasive neuronal changes irrespective of neuronal subtype, we looked at 85 pathways which were common to all neuron-identified clusters: 0, 2, 4, 5, 7, 9, 10, 11, 12, and 14. This analysis revealed activation of the DDR in all neuron subtypes, and in particular several pathways involved in regulation of nucleotide levels were enriched. Indeed, activation of the DDR leads to an increase in nucleotide pools to reduce genotoxic stress, provide resources for repair, and escape potential senescence induction (Moretton and Loizou, 2020). In addition, neurons showed evidence of oxidative phosphorylation, mTOR signaling, selective autophagy, interaction with the innate immune system, proteasome degradation, and cell stress signals. Notably, neuronal clusters also showed enrichment for pathways involved in the initiation of proteinopathies including Parkinson’s disease and amyloid fiber formation. Collectively, the shared enriched pathways across all neuronal clusters provide evidence that events leading to proteinopathy are established early and potentially regulated by the DDR. To identify how different subtypes of neurons may respond to these common signals, we next identified enriched pathways unique to each neuronal cluster. On average, 11.2% of the enriched pathways were unique to a cluster, with cluster 11 showing the most unique signature of 44.0% (Figure 6E). A small subset of the GSEA for neuronal clusters has been visualized in Figure 6F, highlighting features of cellular senescence and features enriched in only a subset of neuronal subtypes. Pathways unique to each neuronal subcluster demonstrate how different subsets of neurons uniquely respond to senescent-like and genotoxic cues and provide a functional differentiation of cells that become hyperexcitable or pro-inflammatory (Figure 6F).

Cluster 0 was identified as excitatory telencephalon projecting neurons. These cells were uniquely enriched for 48 pathways compared to all other neuronal cell clusters. These pathways were involved in cell stress and DNA damage signaling, senescence, the SASP, proteinopathy, and activation of the immune system. In particular, signal transduction stimulated by angiotensin II was enriched in these cells, which has recently been shown to cause DNA damage and senescence (Herbert et al., 2008). Several features of the SASP were enriched such as IL1-β production, Senescence_SASP, production of tumor necrosis factor (TNF), and the PI3K pathway. A key feature of this cluster was enrichment of pathways involved in proteinopathy including pathways for the regulation of tau-protein kinase activity, the unfolded protein response, and Alzheimer’s disease. The unfolded protein response is another stimulus leading to DNA damage induced senescence, conceivably representing a positive feedback loop. Pathways involved in activation of the innate immune system were also evident in this cluster.

Cluster 2 was identified as multiple types of neurons including di- and mesencephalon neurons, cholinergic neurons, and telencephalon interneurons and was enriched for 32 pathways compared to all other neuronal clusters. These unique pathways predominantly reflected the DDR, specifically the global genomic nucleotide excision repair, dysregulation of the postsynaptic density structure and neurotransmission. In addition, unique pathways reflected endoplasmic reticulum stress and altered metabolism of rRNA, insulin, and ATP production.

Cluster 4 was identified as inhibitory telencephalon projecting neurons and was enriched for 74 pathways compared to all other neuronal clusters. Of these unique pathways, 15 involved altered metabolism including positive regulation of ATP production and positive regulation of nucleotides. This alteration in metabolism may reflect increased energy expenditure associated with increased production of pro-inflammatory factors in the SASP and mounting a response against genotoxic stress. Indeed, pathways uniquely enriched in this cluster were consistent with SASP signaling including terms such as “inflammatory response,” “IL-6 signaling pathway,” and “regulation of NF-kappaB signaling” and the SCAP PI3K-Akt pathway. Other enriched terms indicated a senescence-like phenotype including signaling by NOTCH2, which has been shown to induce senescence in neural stem cells via the induction of cell-cycle arrest (Hoare and Narita, 2017), and the AP-1 pathway, which has been shown to be involved in a reversible state of senescence in response to stimuli such as cytokines, growth factors, and stress signals (Martínez-Zamudio et al., 2020; Zumerle and Alimonti, 2020). The enrichment of these two pathways in particular suggest that this cluster of neurons may be receiving paracrine signals from the microenvironment, from SASP factors such as cytokines and growth factors, and acquiring a senescent-like phenotype. Dysregulation of neurotransmission was evident in this cluster with downregulation of terms involved in excitatory postsynaptic potential. Interestingly, this cluster showed enrichment of pathways involved in estrogen signaling through nuclear receptors, indicating that there may be a role of sex hormones in the senescent-like response of neurons to genotoxic stress.

Cluster 5 was identified as excitatory telencephalon projecting neurons and uniquely enriched for 64 pathways compared to all other neuronal clusters. Pathways consistent with DNA damage and the response to stress were upregulated, alongside metabolic reprogramming and presentation of SCAPs and SASP factors. This cluster showed downregulation of pathways consistent with neurotransmission, including regulation of action potential, glutamate receptor signaling pathway, and synapse assembly, indicating a decrease in neurotransmission in this cluster of cells.

Cluster 7 was identified as telencephalon interneurons and uniquely enriched for 38 pathways compared to all other neuronal clusters. Neurotransmitter signaling, particularly via acetylcholine and norepinephrine, was upregulated in this cluster, in addition to inflammatory signaling through TGF-β, secretion by cell, and transportation and localization of lipids. Similarly to cluster 4, this cluster showed enrichment of the late estrogen response and regulation of hormone levels, perhaps indicating a role of sex hormones in the neuronal response to oxidative stress.

Cluster 9 was identified as telencephalon interneurons and uniquely enriched for 30 pathways compared to all other neuronal clusters. Importantly this cluster showed upregulation of the c-Myc signaling pathway and its downstream targets, which has been previously shown in models of brain trauma, stroke, and Alzheimer’s disease (Lee et al., 2011). c-Myc activation in neurons is associated with cell death and cognitive deficits (Lee et al., 2009). This cluster also showed downregulation of several pathways involved in the regulation of gene silencing by miRNAs, suggesting increased transcriptional activation, aberrant miRNA signaling and mRNA splicing, attributes frequently reported in neurodegenerative brains (Maciotta et al., 2013; Li et al., 2021). This cluster of neurons may therefore be undergoing neurodegenerative processes in response to the cell stress signals common to all neuronal clusters as discussed above.

Cluster 10 was identified as dentate gyrus granule neurons and uniquely enriched for 10 pathways compared to all other neuronal clusters. These pathways reflected integrin signaling, metabolism of lipids and steroids, and catabolism of amyloid precursor protein.

Cluster 11 was identified as immature neurons (non-glutamatergic neuroblasts and olfactory inhibitory neurons) and uniquely enriched for 173 pathways compared to all other neuronal clusters. Many of the enriched pathways in this cluster were reflective of increased neurotransmitter signaling, particularly glutamate-mediated signaling through NMDA receptors, acetylcholine signaling through muscarinic receptors 1 and 3, release of glutamate, and alterations in ion transport. Evidence of modification of the postsynaptic structure and hyperexcitability are present, including the trafficking of new AMPA receptors. In addition to changes in cell-cell signaling pathways, the CCR3 pathway was upregulated, which has been identified as a possible driver of viral-induced dementia (van der Meer et al., 2000) and a key mediator of neuronal death (Zhang et al., 2016). This cluster may therefore be responding to the genotoxic and senescent-like signals with hyper-excitability, which has previously been shown to occur in an accelerated senescence mouse model (Nakanishi et al., 1998) and aging (Simkin et al., 2015). Indeed, neuronal hyperexcitability has been associated with cognitive impairment and may be considered an indicator of disease progression from mild cognitive impairment to neurodegenerative disease (Dickerson et al., 2005; Bakker et al., 2012).

Cluster 12 was identified as excitatory telencephalon projecting neurons and uniquely enriched for 56 pathways compared to all other neuronal clusters. Signaling through p38MAPK was enriched in this cluster, including enrichment of ERK targets and nuclear events mediated by MAP kinases. Downregulation of dendritic spine organization and the excitatory postsynaptic potential were evident, suggesting dysfunctional neurotransmission in this cluster.

Cluster 14 was identified as di- and mesencephalon neurons and uniquely enriched for 63 pathways compared to all other neuronal clusters. This cluster was enriched for several pathways involved in the sensing of genotoxic stress including the p53-dependent DNA damage response, and degradation of proteins required for the cell cycle. Consistent with this, the SASP was upregulated in this cluster with terms such as “SASP,” “regulation of acute inflammatory response,” and “positive regulation of cellular response to TGFβ stimulus” enriched. Accompanied by these changes were metabolic reprogramming, activation of the innate immune system, and lamellipodium reorganization consistent with senescence-associated morphological alterations.

Cluster 15 was identified as di- and mesencephalon neurons and uniquely enriched for 46 pathways compared to all other neuronal clusters. A key feature of this cluster was the enrichment of pathways involved in neurotransmission, particularly activation of kainite receptors, response to acetylcholine, glutamate secretion, and catecholamine secretion. This was accompanied by activation of the innate immune response, oxidative stress signals, altered metabolism, and dysregulation of actin filament polymerization.

From this analysis, we have identified that all neuronal clusters show at least one core feature of senescence. p38MAPK signaling was evident in several neuronal clusters, along with high levels of SASP such as IL6, IL8, NFKB, and growth factor signaling. These clusters also tended to show elevation of SCAPs such as P13K/Akt/Metabolic, p53/p21/Serpine, BCL-2 family, HSP90, and HIF1α (Kirkland et al., 2017). Pronounced metabolic reprogramming was evident in the neuronal clusters, such as autophagy, increased glycolysis, and altered mitochondrial metabolism. These mechanisms have previously been identified as features of senescence and have been argued to reflect cells’ energetic adaption to their novel secretory phenotype (Soto-Gamez et al., 2019).

#### Glial and vascular cells show deoxyribonucleic acid damage response and initiation of senescence pathways

Clusters identified as glial cells, including astrocytes, oligodendrocytes, immune cells and vascular cells, were examined for enriched senescence pathways next. A total of 161 common pathways were enriched in all glial cell types (Figure 6G) with some of the unique features of each cell type noted (summary of findings in glial cells visualized in Figure 6H). Pathways involved in cell stress signals and DNA damage pathways were enriched, including cell response to stress, p21-dependent cell cycle checkpoints, and nonsense-mediated decay. Evidence of pathways involved in the response to viral infection were enriched despite absence of infection in these mice. Similar to neurons, glial cells also showed enrichment in pathways involved in energy production via glycolysis and nucleotide synthesis, indicating the cells are mounting a robust DNA damage response involving metabolic reprogramming to counter the damage. Other pathways enriched included protein translation and processing, ROBO/SLIT signaling, and pathways involved in the initiation of proteinopathy including Huntington disease, amyloid fiber formation, and Parkinson’s disease. Together this suggests that glial cells, like neurons, experience extensive genotoxic stress and exhibit a collective response involving metabolic reprogramming to synthesize nucleotides, cell cycle arrest by way of p16 and p21, and the emergence of proteinopathy-consistent pathways (Figure 6H).

In astrocyte-identified clusters, 63 pathways were uniquely enriched compared to other glial cell types encompassing the DNA damage response, astrogliosis, synaptic regulation, SASP, glucose signaling, and autophagy. Astrogliosis was indicated by enrichment of NFAT/calcineurin signaling, which is considered critical for astrocyte activation (Furman and Norris, 2014; Kipanyula et al., 2016) and Rac1 signaling that has recently been shown to control astrogliosis in the context of brain injury (Ishii et al., 2017). Dysregulation of synapses by astrocytes was evident in these clusters, indicated by enrichment of pathways involved in neurotransmitter release cycles, neurotransmitter uptake, retrograde signaling, synaptic vesicle recycling, and regulation of glutamatergic transmission. This is consistent with findings showing that senescent astrocytes lead to glutamate toxicity by means of dysregulation of neuronal synapses (Limbad et al., 2020). Indeed, astrocytes also showed features of the SASP such as interleukin signaling and G-CSF (Coppé et al., 2010). Additionally, metabolism was affected, appearing as altered metal iron homeostasis, a feature of Alzheimer’s disease (Isaev et al., 2020) and increased glucose metabolism and insulin-glucose signaling, a feature of senescence (Wang et al., 2022). Indeed, the latter is closely linked to senescence-associated autophagy (Rajendran et al., 2019) an effect seen in this cell type.

In oligodendrocyte-identified clusters 186 pathways were uniquely enriched compared to other glial cell types. A persistent DNA damage response was evident in this cell type, in particular global nucleotide excision repair, cell stress responses, and regulation of apoptosis in response to DNA damage. Several features of cellular senescence were present, including SASP features such as p38MAPK and interleukin signaling, and SCAPs HIF1α, HSP90, p53, and PI3K/Akt. Anti-apoptotic mechanisms in response to DNA damage and metabolic reprogramming were also supportive of a senescent phenotype. Activation of the innate immune system and signaling through extracellular membrane receptors was enriched in oligodendrocytes, suggesting cell-cell interactions between oligodendrocytes and immune cells and perhaps signaling from SASP factors in the extracellular environment. Dysregulation of actin dynamics was evident, suggesting alterations in myelination (Brown and Macklin, 2020).

In immune cell-identified clusters 85 pathways were uniquely enriched compared to other non-neuronal cell types. Interestingly, enrichment of muscarinic acetylcholine receptor signaling was seen in this cell population, consistent with reports of a subpopulation of microglia in AD and stroke which express functional muscarinic receptors (Pannell et al., 2016). Several features of microglial activation were present, including increased lipid metabolism, mitophagy, and an enhanced inflammatory response. Other changes seen in this cell type include extracellular matrix degradation, altered protein metabolism, alternative mRNA splicing, and increased ncRNA processing. Clear immune cell-neuronal interactions were present, including the positive regulation of neuron death and response to axonal injury. Because the distinction between activated microglia and senescent microglia is not clear due to overlaps in inflammatory features, growth factor signaling, and metabolic changes (Harry, 2013), we cannot conclude whether these microglia are senescent or activated.

In vascular cells 29 pathways were uniquely enriched compared to all other cell types. These cell types showed some evidence that SASP factors were signaling to surrounding cell types through the extracellular environment. Vascular cells were enriched for Integrin 3 signaling, which has previously been shown to induce cellular senescence through activation of the TGFβ pathway (Rapisarda et al., 2017). Integrins are cell surface molecules which can be activated by several SASP factors including growth factors, proteases, cytokines, or pathogens (Borghesan and O’Loghlen, 2017). In congruence, these cells were enriched for IL-6 production, tumor necrosis factor production, chemokine production, and growth factor signaling. Moreover, these cells showed a strong response to stress, and positive regulation of amyloid precursor protein catabolism alongside altered mRNA splicing.

### Treatment with senolytic drug ABT263 reduces senescence in males but not females

**FIGURE 7:**
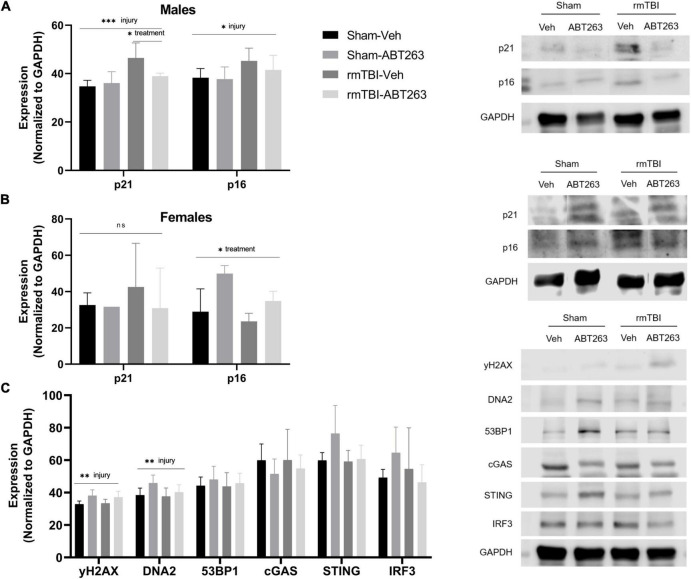
Treatment with senolytic drug ABT263 reduces senescence in males, but not females, following rmTBI. Western blot analysis revealed that ABT263 treatment 1 week after injuries followed by sacrifice 1 week later (total of 2 weeks post-injuries) significantly reduced expression of p21 in male rmTBI mice **(A)** (*p* = 0.02, two-way ANOVA), which was significantly increased by injury (*p* = 0.0003, main effect of injury, two-way ANOVA). p16 was also significantly increased by injury (*p* = 0.02, main effect of injury, two-way ANOVA) in the males **(A)** and was only partially reduced by ABT263 treatment, although this did not reach statistical significance. In contrast, females showed no significant differences in p21 protein levels by this timepoint **(B)**. Interestingly, p16 protein levels significantly increased with ABT263 treatment in female mice (*p* = 0.03, main effect of treatment, two-way ANOVA). ABT-263 treatment did not significantly reduce markers of DNA damage nor cGAS-STING signaling **(C)** in male mice, indicating the remaining presence of DNA damage and inflammatory signal. Error bars represent standard deviation with *n* = 4 per group. Statistical significance is marked as *p < 0.05, ^**^p < 0.01, ^***^p < 0.001. ns, not significant.

Male and female rmTBI and sham mice were given either vehicle or ABT263 1 week following injury, and sacrificed 1 week later to assess the effect on DNA damage-induced senescence pathways in a sex-segregated manner (Figure 7). In the males, western blot analysis revealed that treatment with ABT263 significantly reduced expression of p21 (*p* = 0.02, two-way ANOVA), which had been significantly increased by injury (*p* = 0.0003, main effect of injury, two-way ANOVA; Figure 7A). In the males, p16 was significantly increased by rmTBI (*p* = 0.02, main effect of injury, two-way ANOVA), although this was only partially restored after ABT263 treatment and did not reach statistical significance (Figure 7A). In the females, there was no significant effect of injury or treatment on p21 by this time point (Figure 7B). Interestingly, p16 increased significantly with ABT263 treatment specifically in the females (*p* = 0.03, main effect of treatment, two-way ANOVA; Figure 7B).

To assess whether reducing senescence with ABT263 impacted downstream signaling pathways, we further assessed the male mice. Although not significant (two-way ANOVAs), ABT263 treatment partially reduced expression of DNA2, STING, and IRF3 (Figure 7C). There was no effect on γH2AX or 53BP1, indicating that DNA damage and pro-inflammatory signaling remain despite senolytic treatment (Figure 7C).

### Treatment with senolytic drug ABT263 improves memory after rmTBI

**FIGURE 8:**
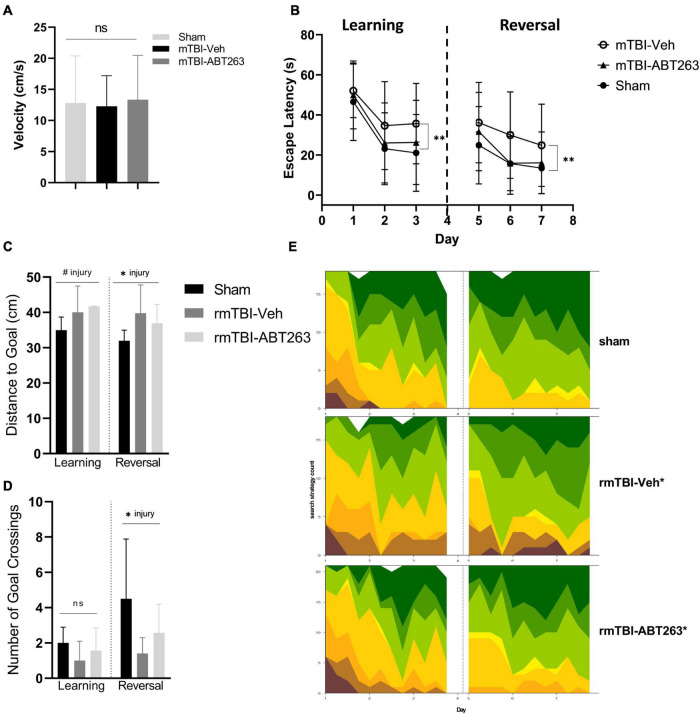
Treatment with senolytic drug ABT263 improves cognitive dysfunction after rmTBI. Mice given rmTBI followed by vehicle or ABT263 treatment were tested in MWM compared to shams. Swim speed did not significantly differ between groups **(A)**. In the learning and reversal learning trials, rmTBI-veh mice had a significantly higher escape latency compared to both shams and rmTBI-ABT263 mice (*p* < 0.001, main effect of injury treatment group) for both learning and reversal learning trials **(B)**. In the probe tests, treatment did not significantly affect the distance to the goal **(C)** nor number of goal crossings **(D)**. However, injury regardless of treatment significantly increased the distance to the goal **(C)** (*p* < 0.05, main effect of injury) and number of goal crossings **(D)** (*p* < 0.05, main effect of injury). Machine learning classifying of search strategies using RTrack **(E)** confirmled that sham and rmTBI-ABT263 mice used significantly more goal-oriented strategies compared to rmTBI-veh mice (*p* < 0.0001, *x*^2^ = 29.47, Chi square). Error bars represent standard deviation with an *n* of 8 per group. Statistical significance is marked as ^#^*p* < 0.1, *p < 0.05, ^**^p < 0.01, ^***^p < 0.001. ns, not significant.

One week after treatment with ABT263 (2 weeks after injury), male mice were tested on the MWM for analysis of spatial memory and executive functioning to assess whether reducing senescent cell burden has behavioral repercussions in a cause-and-effect relationship (Figure 8). Swim speed did not significantly differ between groups (Figure 8A). In both the learning and reversal learning trials, rmTBI-veh mice had a significantly higher escape latency compared to both shams and rmTBI-ABT263 mice (*p* < 0.001, main effect of injury-treatment group, three-way ANOVA; Figure 8B). The probe trial did not show statistically significant differences in distance to the goal (Figure 8C) nor number of goal crossings (Figure 8D), as the ABT263 treated rmTBI mice performed in between sham mice and vehicle-treated rmTBI mice. These data indicate that ABT263 treatment significantly improves cognition and executive functioning after brain injury, but that a small deficit remains, complementary to the molecular data presented in the previous section. The corrective effect of ABT263 is evidence in support of senescence playing a role in brain dysfunction following rmTBI.

Search strategy analysis further confirmed the presence of cognitive and executive dysfunction in injured mice compared to shams (Figure 8E). Overall, sham and rmTBI-ABT treated mice used more goal-oriented strategies and less non-goal-oriented strategies compared to rmTBI-vehicle treated mice (*p* < 0.0001, χ^2^ = 29.47, Chi-Square analysis). In the learning paradigm, from training day one to training day three sham mice and ABT263 treated injured mice increased the use of goal-oriented strategies by 50.0 and 39.6%, respectively, whereas vehicle treated injured mice only increased their use by 30.2%. Similarly, in the reversal learning paradigm from training days five to seven sham mice and ABT263 treated injured mice increased the use of goal-oriented strategies by 17.1 and 28.6%, respectively, whereas vehicle treated injured mice only increased by 16.9%. Therefore, it appears that in reversal learning the ABT263 treated group may have out-performed shams in the use of goal-oriented strategies to find the platform. Together, these data indicate that deficits in spatial memory and executive function after rmTBI can be reversed by treatment with ABT263.

## Discussion

Cellular senescence is a complex molecular cascade which underlies many geriatric diseases and CNS disorders (Chinta et al., 2018; Vazquez-Villaseñor et al., 2020; Guerrero et al., 2021). Evolutionarily speaking, senescence has emerged as a beneficial pathway in non-diseased contexts. For example, senescent cells assist in tissue remodeling during development (Storer et al., 2013), childbirth (Cha and Aronoff, 2017), wound repair (Wilkinson and Hardman, 2020), and are a defense against cancer (Yang et al., 2021). However, when senescent cells are persistent, they become deleterious and have been shown to be a causative agent in disease onset. For instance, transplanting senescent cells into middle aged mice causes a significant increase in frailty and death from all causes (Xu et al., 2018). Senescence is predominantly characterized by permanent cell cycle arrest and upregulation of anti-apoptotic SCAP networks, and some senescent cells express the SASP leading to tissue damage, age-related disease, and reduced resilience to insults (Childs et al., 2017; Kumari and Jat, 2021). These cells can cause the activation of cGAS-STING in response to DNA damage, and this further exacerbates SASP through the activity of interferon-1 (Yang et al., 2017). Our previous work in both humans and mice (Schwab et al., 2019, 2021) alongside studies by others (Ritzel et al., 2019; Tominaga et al., 2019) has shown that senescent cells accumulate after brain injury and may contribute to a pathophysiological mechanism driving injury-related brain dysfunction.

Here we have shown that DNA damage-induced cellular senescence, accompanied by cGAS-STING type-I interferon signaling is an early event after rmTBI, evident 1 week after the final injury. Using scRNAseq, we have shown that senescent-like expression changes are apparent in both glial cells and neurons. These findings support previous studies by our lab and others showing evidence of DNA damage and cellular senescence after rmTBI (Ritzel et al., 2019; Schwab et al., 2019, 2021; Tominaga et al., 2019). The idea that neurons can enter a state of senescence has been controversial, as the phenotype has previously been defined by cell-cycle arrest (Blagosklonny, 2006). However, it has been shown using immunohistochemistry that neurons upregulate cell-cycle control proteins p21 and p16 in response to brain injury (Tominaga et al., 2019), and similarly these senescence markers are also elevated in neurons of ALS brains in the early stages of disease (Vazquez-Villaseñor et al., 2020). Consistent with these previous findings, this study has used scRNAseq to show that neurons are indeed enriched for pathways involved in cellular senescence including the DNA damage response, cell-cycle arrest mediated by p53, p21, and p16, p38MAPK signaling, and production of pro-inflammatory chemokines and cytokines consistent with the SASP. Using this technique, we have expanded on these previous findings to identify changes which may have repercussions on overall brain function. Several clusters of neurons showed enrichment of pathways indicating altered neurotransmission, hyperexcitability, structural changes to the synaptic density, interactions with the brain’s innate immune system, metabolic and morphological shifts, and alterations in the expression of ion channels involved in regulating membrane potential. Similarly, dysregulation of neurotransmission was evident in astrocytes with downregulation of pathways involved in glutamate uptake and vesicle recycling. These findings are consistent with a cell culture study showing that astrocyte senescence leads to reduced expression of glutamate and potassium transporters, thereby leading to neuronal death via excitotoxicity (Limbad et al., 2020). In general, these findings may reflect the clinical presentation of some individuals with TBI who have epileptic seizures (Englander et al., 2014), ADHD (Stojanovski et al., 2019a), and behavioral dysregulation (Andrews et al., 1998). Interestingly, most cell types were enriched for pathways consistent with proteinopathy and neurodegenerative diseases including AD, PD, amyloid fiber formation, iron uptake, alpha-synuclein signaling, and the unfolded protein response. This data therefore suggests that alongside DNA damage-induced senescence, pathways involved in the emergence of proteinopathy have an early onset after rmTBI. Indeed, mTBI is associated with proteinopathy not only in neurons but in glial cells as well (Bieniek et al., 2021).

We then tested the use of ABT263, a senolytic drug specifically targeting senescent cells for apoptosis via inhibition of the BCL-2 SCAP network, in rescuing aberrant molecular markers and neurobehavioral changes seen at 1-week post-injury. In males, administration of ABT263 significantly improved performance in the MWM, suggesting that the use of senolytics is useful for reversing functional deficits after mTBI, although a small deficit remained as seen in the probe test. Molecularly, ABT263 significantly reduced expression of senescence proteins, and only modestly reduced markers of the DDR and cGAS-STING pathways without reaching significance. DNA damage markers γH2AX and 53BP1 remained elevated in ABT263 treated rmTBI mice. These findings suggest that although senolytic treatment improves behavioral outcomes and some aspects of aberrant molecular signatures for senescence and DNA damage response post-mTBI, the source of these deficits, namely DNA damage, is not eliminated nor are the downstream pro-inflammatory signaling cascades. Importantly, this study administered ABT263 systemically. This has been considered an optimal approach over local administration, as senescence spreads from cell to cell and local accumulation of senescence can quickly become systemic (Gonzalez-Meljem et al., 2018).

Senolytic treatment with ABT263 in the context of rmTBI has several advantages and limitations. A key advantage of senolytic drugs is that they target senescent cells that become established and maintained in the long term, as such they could in theory be effective long after the brain injury has occurred. Moreover, senolytic treatment can be administered intermittently rather than continuously, thus potentially minimizing toxicity effects and improving patient compliance in a clinical setting. This is a significant advantage in comparison to potential emergency therapeutics that must be administered immediately post-injury, due to their diminishing efficacy after the critical time window. Indeed, use of senolytic drugs on mouse models have shown to be effective in chronic long-term conditions with an established pathology, such as AD (Zhang et al., 2019). However, the use of senolytics also comes with inherent limitations. Senescent cells often use more than one SCAP network to evade self-destruction from pro-apoptotic SASP factors, thus targeting more than one SCAP network would be beneficial in improving specificity and targeting more senescent cells (Ovadya and Krizhanovsky, 2018). ABT263 only inhibits the BCL-2 family SCAP network and has been shown to have off-target effects on non-senescent cells specifically platelets and immune cells (Wilson et al., 2010; Mahfoudhi et al., 2016) while being ineffective against senescent cells expressing multiple redundant SCAP networks. Using drugs that target more than one SCAP network simultaneously would increase specificity toward senescent cells, reduce off-target effects on non-senescent cells, and thereby reduce side-effects (Kirkland and Tchkonia, 2020). This study has shown that neurons express senescent gene signatures and therefore may be targets of senolytic drugs. The long-term effects of eliminating functional groups of neurons that cannot repopulate remain unclear, however, it is reasonable to hypothesize that it may eventually cause disease or brain dysfunction. To address these limitations, several therapies against senescent cells are being tested in preclinical models. Some of these promising new therapies include nanoparticles that, when taken in by senescent cells, lead to the induction of apoptosis via the delivery of toxic cargos, vaccine delivered treatments, and treatments geared toward the pro-inflammatory factors associated with the SASP (Weiland et al., 2014; Muñoz-Espín et al., 2018; Li et al., 2019). The effectiveness and safety of such compounds are yet to be determined. In addition, based on novel findings from this study, sex differences in the mechanism of DNA damage-induced cellular senescence render it possible that optimal treatment targets may be sex-specific.

Molecular data was either analyzed using sex as a variable, or in the case of senolytic in a sex-segregated manner, highlighting key differences supporting the notion that senolytic drugs may not be a blanket solution for all patients. Female rmTBI mice displayed elevated levels of DNA damage in the form of DSBs, oxidative damage, and R-Loops compared to males, however, both males and females showed elevated expression of senescent markers p21 and p16. This data indicates that despite female mice having elevated levels of DNA damage, they do not seem to have a significantly higher expression of this senescent signature, indicating that females may have a higher tolerance for genotoxic stress than males. In the senolytic mice, a surprising trend emerged: male mice significantly reduced expression of senescent markers in response to treatment, but female mice showed the opposite response. Indeed, administering ABT263 increased expression of p16 in both sham and rmTBI female mice, but not their male counterparts. This indicates that perhaps ABT263 may not be a beneficial drug in the post-rmTBI female. Importantly, by this time point (2 weeks following injuries), p21 and p16 are still elevated in male rmTBI mice compared to shams, but levels appear to have stabilized in the female mice such that there is no main effect of injury remaining. It is therefore possible that despite the higher burden of DNA damage, female mice are able to promote cell-cycle arrest and repair in the first week following injury, avoiding entering a permanent state of cellular senescence unlike their male counterparts. Indeed, ABT263 increasing expression of senescence proteins in female rmTBI mice at this time point indicates that in a non-senescent context these drugs may be harmful, perhaps due to the off-target effects described above, and their safety examined further. Thus, subsequent protein assays and behavioral assays were performed in males, to assess whether the reduced senescent cell burden was associated with improved cognition. A limitation of this study is therefore the inclusion of only male mice in the senolytic behavioral assays.

The mechanisms driving the sex differences detected in this study remain exploratory, however, several factors may be involved such as loss of estrogen-related neuroprotection in the immediate period following injury and higher tolerance of genotoxic stress in females in general. Studies in both humans (Dimopoulou et al., 2004) and mice (Russell et al., 2018) have shown dysregulation of the hypothalamic-pituitary-adrenal axis following brain injury in which estrogen precursor levels in the brain significantly drop in the acute period after injury. Indeed, in a weight drop model of mTBI the estrogen precursor dehydroepiandrosterone (DHEA) was found to be significantly reduced in the brain in the 2-week period following mTBI (Lopez-Rodriguez et al., 2015). This effect is thought to be observed predominantly in females, with levels in male brains staying relatively unchanged. Since estrogen is neuroprotective against genotoxic stress (Brotfain et al., 2016; Raghava et al., 2017; Duncan, 2020) and administrating estrogen has been shown to improve neurological impairment after TBI in female mice (Lu et al., 2018), it is reasonable to suggest that female mice are showing higher levels of DNA damage and cellular senescence in this 1 week period due to a drop in estrogen-derived neuroprotection. This study did not directly assess hormone levels, and therefore no conclusions can be made regarding the role of estrogen in rmTBI in this study due to this limitation. We propose that future studies on rmTBI use both male and female animals and/or patients, and present sex-segregated data so that this question can be explored further.

## Conclusion

This study has provided evidence supporting a causal link between the accumulation of senescent neurons and glial cells and the pathogenesis of rmTBI-related brain dysfunction. This study has further demonstrated that post-rmTBI administration of the senolytic drug ABT263 reduces senescence in males but not females, and improves behavioral outcomes in males after rmTBI. These results suggest sex-dependent effects of DNA damage-induced senescence, and by extension senolytic drugs. These novel findings prompt us to suggest that future therapies targeting cellular senescence after brain injury should be personalized and targeted comprehensively by using multiple molecular targets, and that all future studies on brain injury should include sex as a variable studied.

## Data availability statement

The scRNAseq raw data can be found in online repositories. The names of the repository/repositories and the GEO accession numbers GSM6680914, GSM6680915, GSM6680916, and GSM6680917 can be found in the article/Supplementary material. Further inquiries can be directed to the corresponding author.

## Ethics statement

This animal study was reviewed and approved by Centre for Phenogenomics Animal Care Committee.

## Author contributions

NS: study design, collection and analysis of all data including statistical tests, animal experiments and handling, and manuscript writing. DT: collection and analysis of statistical data and input on manuscript. EL: analysis of western blot data and input on manuscript. BI: single-cell RNA sequencing data analysis and input on manuscript. GB: single-cell RNA sequencing supervision. L-NH: study design, acquisition of study funding, input on manuscript, and supervision. All authors contributed to the article and approved the submitted version.

## References

[B1] Alquicira-Hernandez J. Sathe A. Ji H. P. Nguyen Q. Powell J. E. (2019). scPred: Accurate supervised method for cell-type classification from single-cell RNA-seq data. *Genome Biol.* 20:264. 10.1186/s13059-019-1862-5 31829268 PMC6907144

[B2] Andrews T. K. Rose F. D. Johnson D. A. (1998). Social and behavioral effects of traumatic brain injury in children. *Brain Inj.* 12 133–138. 10.1080/026990598122755 9492960

[B3] Bakker A. Krauss G. L. Albert M. S. Speck C. L. Jones L. R. Stark C. E. (2012). Reduction of hippocampal hyperactivity improves cognition in amnestic mild cognitive impairment. *Neuron* 74 467–474. 10.1016/j.neuron.2012.03.023 22578498 PMC3351697

[B4] Barrett J. P. Knoblach S. M. Bhattacharya S. Gordish-Dressman H. Stoica B. A. Loane D. J. (2021). Traumatic brain injury induces cGAS activation and type I interferon signaling in aged mice. *Front. Immunol.* 12:710608. 10.3389/fimmu.2021.710608 34504493 PMC8423402

[B5] Bieniek K. F. Cairns N. J. Crary J. F. Dickson D. W. Folkerth R. D. Keene C. D. (2021). The second NINDS/NIBIB consensus meeting to define neuropathological criteria for the diagnosis of chronic traumatic encephalopathy. *J. Neuropathol. Exp. Neurol.* 80 210–219. 10.1093/jnen/nlab001 33611507 PMC7899277

[B6] Blagosklonny M. V. (2006). Cell senescence: Hypertrophic arrest beyond the restriction point. *J. Cell Physiol.* 209 592–597. 10.1002/jcp.20750 17001692

[B7] Borghesan M. O’Loghlen A. (2017). Integrins in senescence and aging. *Cell Cycle* 16 909–910. 10.1080/15384101.2017.1316573 28459356 PMC5462089

[B8] Brotfain E. Gruenbaum S. E. Boyko M. Kutz R. Zlotnik A. Klein M. (2016). Neuroprotection by estrogen and progesterone in traumatic brain injury and spinal cord injury. *Curr. Neuropharmacol.* 14 641–653. 10.2174/1570159x14666160309123554 26955967 PMC4981744

[B9] Brown T. L. Macklin W. B. (2020). The actin cytoskeleton in myelinating cells. *Neurochem. Res.* 45 684–693. 10.1007/s11064-019-02753-0 30847860 PMC6732044

[B10] Cha J. M. Aronoff D. M. (2017). A role for cellular senescence in birth timing. *Cell Cycle* 16 2023–2031. 10.1080/15384101.2017.1371888 28873006 PMC5731422

[B11] Childs B. G. Gluscevic M. Baker D. J. Laberge R.-M. Marquess D. Dananberg J. (2017). Senescent cells: An emerging target for diseases of ageing. *Nat. Rev. Drug Discov.* 16 718–735. 10.1038/nrd.2017.116 28729727 PMC5942225

[B12] Chinta S. J. Woods G. Demaria M. Rane A. Zou Y. McQuade A. (2018). Cellular senescence is induced by the environmental neurotoxin paraquat and contributes to neuropathology linked to Parkinson’s disease. *Cell Rep.* 22 930–940. 10.1016/j.celrep.2017.12.092 29386135 PMC5806534

[B13] Coppé J.-P. Desprez P.-Y. Krtolica A. Campisi J. (2010). The senescence-associated secretory phenotype: The dark side of tumor suppression. *Annu. Rev. Pathol.* 5 99–118. 10.1146/annurev-pathol-121808-102144 20078217 PMC4166495

[B14] Decout A. Katz J. D. Venkatraman S. Ablasser A. (2021). The cGAS-STING pathway as a therapeutic target in inflammatory diseases. *Nat. Rev. Immunol.* 21 548–569. 10.1038/s41577-021-00524-z 33833439 PMC8029610

[B15] Dickerson B. C. Salat D. H. Greve D. N. Chua E. F. Rand-Giovannetti E. Rentz D. M. (2005). Increased hippocampal activation in mild cognitive impairment compared to normal aging and AD. *Neurology* 65 404–411. 10.1212/01.wnl.0000171450.97464.49 16087905 PMC4335677

[B16] Dimopoulou I. Tsagarakis S. Kouyialis A. T. Roussou P. Assithianakis G. Christoforaki M. (2004). Hypothalamic-pituitary-adrenal axis dysfunction in critically ill patients with traumatic brain injury: Incidence, pathophysiology, and relationship to vasopressor dependence and peripheral interleukin-6 levels. *Crit. Care Med.* 32 404–408. 10.1097/01.CCM.0000108885.37811.CA 14758155

[B17] Diniz B. S. Reynolds Iii C. F. Sibille E. Bot M. Penninx B. W. J. H. (2019). Major depression and enhanced molecular senescence abnormalities in young and middle-aged adults. *Transl. Psychiatry* 9:198. 10.1038/s41398-019-0541-3 31434875 PMC6704136

[B18] Duncan K. A. (2020). Estrogen formation and inactivation following TBI: What we know and where we could go. *Front. Endocrinol.* 11:345. 10.3389/fendo.2020.00345 32547495 PMC7272601

[B19] Englander J. Cifu D. X. Diaz-Arrastia R. (2014). Information/education page. Seizures and traumatic brain injury. *Arch. Phys. Med. Rehabil.* 95 1223–1224. 10.1016/j.apmr.2013.06.002 24862307 PMC4516165

[B20] Fatt M. P. Tran L. M. Vetere G. Storer M. A. Simonetta J. V. Miller F. D. (2022). Restoration of hippocampal neural precursor function by ablation of senescent cells in the aging stem cell niche. *Stem Cell Rep.* 17 259–275. 10.1016/j.stemcr.2021.12.010 35063124 PMC8828532

[B21] Fielder E. von Zglinicki T. Jurk D. (2017). The DNA damage response in neurons: Die by apoptosis or survive in a senescence-like state? *J. Alzheimers Dis.* 60 S107–S131. 10.3233/JAD-161221 28436392

[B22] Finak G. McDavid A. Yajima M. Deng J. Gersuk V. Shalek A. K. (2015). MAST: A flexible statistical framework for assessing transcriptional changes and characterizing heterogeneity in single-cell RNA sequencing data. *Genome Biol.* 16:278. 10.1186/s13059-015-0844-5 26653891 PMC4676162

[B23] Furman J. L. Norris C. M. (2014). Calcineurin and glial signaling: Neuroinflammation and beyond. *J. Neuroinflammation* 11:158. 10.1186/s12974-014-0158-7 25199950 PMC4172899

[B24] George K. K. Heithoff B. P. Shandra O. Robel S. (2022). Mild traumatic brain injury/concussion initiates an atypical astrocyte response caused by blood-brain barrier dysfunction. *J. Neurotrauma* 39 211–226. 10.1089/neu.2021.0204 34806422 PMC8785769

[B25] Gonzalez-Meljem J. M. Apps J. R. Fraser H. C. Martinez-Barbera J. P. (2018). Paracrine roles of cellular senescence in promoting tumourigenesis. *Br. J. Cancer* 118 1283–1288. 10.1038/s41416-018-0066-1 29670296 PMC5959857

[B26] Guerrero A. De Strooper B. Arancibia-Cárcamo I. L. (2021). Cellular senescence at the crossroads of inflammation and Alzheimer’s disease. *Trends Neurosci.* 44 714–727. 10.1016/j.tins.2021.06.007 34366147

[B27] Gupta R. Sen N. (2016). Traumatic brain injury: A risk factor for neurodegenerative diseases. *Rev. Neurosci.* 27 93–100. 10.1515/revneuro-2015-0017 26352199

[B28] Hao Y. Hao S. Andersen-Nissen E. Mauck W. M. Zheng S. Butler A. (2021). Integrated analysis of multimodal single-cell data. *Cell* 184 3573–3587.e29. 10.1016/j.cell.2021.04.048 34062119 PMC8238499

[B29] Harry G. J. (2013). Microglia during development and aging. *Pharmacol. Ther.* 139 313–326. 10.1016/j.pharmthera.2013.04.013 23644076 PMC3737416

[B30] Hegazy Y. A. Fernando C. M. Tran E. J. (2020). The balancing act of R-loop biology: The good, the bad, and the ugly. *J. Biol. Chem.* 295 905–913. 10.1074/jbc.REV119.011353 31843970 PMC6983857

[B31] Herbert K. E. Mistry Y. Hastings R. Poolman T. Niklason L. Williams B. (2008). Angiotensin II-mediated oxidative DNA damage accelerates cellular senescence in cultured human vascular smooth muscle cells via telomere-dependent and independent pathways. *Circ. Res.* 102 201–208. 10.1161/CIRCRESAHA.107.158626 17991883 PMC2861985

[B32] Hoare M. Narita M. (2017). NOTCH and the 2 SASPs of senescence. *Cell Cycle* 16 239–240. 10.1080/15384101.2016.1248730 27764549 PMC5345188

[B33] Iacono W. G. Malone S. M. McGue M. (2003). Substance use disorders, externalizing psychopathology, and P300 event-related potential amplitude. *Int. J. Psychophysiol.* 48 147–178. 10.1016/s0167-8760(03)00052-7 12763572

[B34] Innes B. T. Bader G. D. (2018). scClustViz – single-cell RNAseq cluster assessment and visualization. *F1000Res* 7:ISCB Comm J-1522. 10.12688/f1000research.16198.2 31016009 PMC6456841

[B35] Isaev N. K. Stelmashook E. V. Genrikhs E. E. (2020). Role of zinc and copper ions in the pathogenetic mechanisms of traumatic brain injury and Alzheimer’s disease. *Rev. Neurosci.* 31 233–243. 10.1515/revneuro-2019-0052 31747384

[B36] Ishii T. Ueyama T. Shigyo M. Kohta M. Kondoh T. Kuboyama T. (2017). A novel Rac1-GSPT1 signaling pathway controls astrogliosis following central nervous system injury. *J. Biol. Chem.* 292 1240–1250. 10.1074/jbc.M116.748871 27941025 PMC5270470

[B37] Kipanyula M. J. Kimaro W. H. Seke Etet P. F. (2016). The emerging roles of the calcineurin-nuclear factor of activated T-lymphocytes pathway in nervous system functions and diseases. *J. Aging Res.* 2016:5081021. 10.1155/2016/5081021 27597899 PMC5002468

[B38] Kirkland J. L. Tchkonia T. (2020). Senolytic drugs: From discovery to translation. *J. Intern. Med.* 288 518–536. 10.1111/joim.13141 32686219 PMC7405395

[B39] Kirkland J. L. Tchkonia T. Zhu Y. Niedernhofer L. J. Robbins P. D. (2017). The clinical potential of senolytic drugs. *J. Am. Geriatr. Soc.* 65 2297–2301. 10.1111/jgs.14969 28869295 PMC5641223

[B40] Kumari R. Jat P. (2021). Mechanisms of cellular senescence: Cell cycle arrest and senescence associated secretory phenotype. *Front. Cell Dev. Biol.* 9:645593. 10.3389/fcell.2021.645593 33855023 PMC8039141

[B41] Lee H. Casadesus G. Nunomura A. Zhu X. Castellani R. J. Richardson S. L. (2009). The neuronal expression of MYC causes a neurodegenerative phenotype in a novel transgenic mouse. *Am. J. Pathol.* 174 891–897. 10.2353/ajpath.2009.080583 19164506 PMC2665749

[B42] Lee H.-P. Kudo W. Zhu X. Smith M. A. Lee H. (2011). Early induction of c-Myc is associated with neuronal cell death. *Neurosci. Lett.* 505 124–127. 10.1016/j.neulet.2011.10.004 22005580 PMC3234683

[B43] Li D. McIntosh C. S. Mastaglia F. L. Wilton S. D. Aung-Htut M. T. (2021). Neurodegenerative diseases: A hotbed for splicing defects and the potential therapies. *Transl. Neurodegener.* 10:16. 10.1186/s40035-021-00240-7 34016162 PMC8136212

[B44] Li W. He Y. Zhang R. Zheng G. Zhou D. (2019). The curcumin analog EF24 is a novel senolytic agent. *Aging* 11 771–782. 10.18632/aging.101787 30694217 PMC6366974

[B45] Lim S. Kim T. J. Kim Y.-J. Kim C. Ko S.-B. Kim B.-S. (2021). Senolytic therapy for cerebral ischemia-reperfusion injury. *Int. J. Mol. Sci.* 22:11967. 10.3390/ijms222111967 34769397 PMC8584561

[B46] Limbad C. Oron T. R. Alimirah F. Davalos A. R. Tracy T. E. Gan L. (2020). Astrocyte senescence promotes glutamate toxicity in cortical neurons. *PLoS One* 15:e0227887. 10.1371/journal.pone.0227887 31945125 PMC6964973

[B47] Liu J. C. Granieri L. Shrestha M. Wang D.-Y. Vorobieva I. Rubie E. A. (2018). Identification of CDC25 as a common therapeutic target for triple-negative breast cancer. *Cell Rep.* 23 112–126. 10.1016/j.celrep.2018.03.039 29617654 PMC9357459

[B48] Lopez-Rodriguez A. B. Acaz-Fonseca E. Giatti S. Caruso D. Viveros M.-P. Melcangi R. C. (2015). Correlation of brain levels of progesterone and dehydroepiandrosterone with neurological recovery after traumatic brain injury in female mice. *Psychoneuroendocrinology* 56 1–11. 10.1016/j.psyneuen.2015.02.018 25770855

[B49] Lu H. Ma K. Jin L. Zhu H. Cao R. (2018). 17β-estradiol rescues damages following traumatic brain injury from molecule to behavior in mice. *J. Cell Physiol.* 233 1712–1722. 10.1002/jcp.26083 28681915

[B50] Lun A. T. L. Bach K. Marioni J. C. (2016). Pooling across cells to normalize single-cell RNA sequencing data with many zero counts. *Genome Biol.* 17:75. 10.1186/s13059-016-0947-7 27122128 PMC4848819

[B51] Maciotta S. Meregalli M. Torrente Y. (2013). The involvement of microRNAs in neurodegenerative diseases. *Front. Cell Neurosci.* 7:265. 10.3389/fncel.2013.00265 24391543 PMC3867638

[B52] Mahfoudhi E. Lordier L. Marty C. Pan J. Roy A. Roy L. (2016). P53 activation inhibits all types of hematopoietic progenitors and all stages of megakaryopoiesis. *Oncotarget* 7 31980–31992. 10.18632/oncotarget.7881 26959882 PMC5077990

[B53] Martínez-Zamudio R. I. Roux P.-F. de Freitas J. A. N. L. F. Robinson L. Doré G. Sun B. (2020). AP-1 imprints a reversible transcriptional programme of senescent cells. *Nat. Cell Biol.* 22 842–855. 10.1038/s41556-020-0529-5 32514071 PMC7899185

[B54] Merico D. Isserlin R. Stueker O. Emili A. Bader G. D. (2010). Enrichment map: A network-based method for gene-set enrichment visualization and interpretation. *PLoS One* 5:e13984. 10.1371/journal.pone.0013984 21085593 PMC2981572

[B55] Moretton A. Loizou J. I. (2020). Interplay between cellular metabolism and the DNA damage response in cancer. *Cancers* 12:E2051. 10.3390/cancers12082051 32722390 PMC7463900

[B56] Muñoz-Espín D. Rovira M. Galiana I. Giménez C. Lozano-Torres B. Paez-Ribes M. (2018). A versatile drug delivery system targeting senescent cells. *EMBO Mol. Med.* 10:e9355. 10.15252/emmm.201809355 30012580 PMC6127887

[B57] Nakanishi H. Miyazaki M. Takai N. Wang H. D. Yamamoto T. Watanabe S. (1998). Hyperexcitability of amygdala neurons of senescence-accelerated mouse revealed by electrical and optical recordings in an in vitro slice preparation. *Brain Res.* 812 142–149. 10.1016/s0006-8993(98)00968-8 9813291

[B58] Namjoshi D. R. Cheng W. H. McInnes K. A. Martens K. M. Carr M. Wilkinson A. (2014). Merging pathology with biomechanics using CHIMERA (closed-head impact model of engineered rotational acceleration): A novel, surgery-free model of traumatic brain injury. *Mol. Neurodegener.* 9:55. 10.1186/1750-1326-9-55 25443413 PMC4269957

[B59] Ovadya Y. Krizhanovsky V. (2018). Strategies targeting cellular senescence. *J. Clin. Invest.* 128 1247–1254. 10.1172/JCI95149 29608140 PMC5873866

[B60] Overall R. W. Zocher S. Garthe A. Kempermann G. (2020). Rtrack: A software package for reproducible automated water maze analysis. *bioRxiv* [Preprint]. 10.1101/2020.02.27.967372

[B61] Pannell M. Meier M. A. Szulzewsky F. Matyash V. Endres M. Kronenberg G. (2016). The subpopulation of microglia expressing functional muscarinic acetylcholine receptors expands in stroke and Alzheimer’s disease. *Brain Struct. Funct.* 221 1157–1172. 10.1007/s00429-014-0962-y 25523105

[B62] Paul B. D. Snyder S. H. Bohr V. A. (2021). Signaling by cGAS-STING in neurodegeneration, neuroinflammation, and aging. *Trends Neurosci.* 44 83–96. 10.1016/j.tins.2020.10.008 33187730 PMC8662531

[B63] Pearn M. L. Niesman I. R. Egawa J. Sawada A. Almenar-Queralt A. Shah S. B. (2017). Pathophysiology associated with traumatic brain injury: Current treatments and potential novel therapeutics. *Cell Mol. Neurobiol.* 37 571–585. 10.1007/s10571-016-0400-1 27383839 PMC11482200

[B64] Raghava N. Das B. C. Ray S. K. (2017). Neuroprotective effects of estrogen in CNS injuries: Insights from animal models. *Neurosci. Neuroecon.* 6 15–29. 10.2147/NAN.S105134 28845391 PMC5567743

[B65] Rajendran P. Alzahrani A. M. Hanieh H. N. Kumar S. A. Ben Ammar R. Rengarajan T. (2019). Autophagy and senescence: A new insight in selected human diseases. *J. Cell Physiol.* 234 21485–21492. 10.1002/jcp.28895 31144309

[B66] Rapisarda V. Borghesan M. Miguela V. Encheva V. Snijders A. P. Lujambio A. (2017). Integrin Beta 3 regulates cellular senescence by activating the TGF-β pathway. *Cell Rep.* 18 2480–2493. 10.1016/j.celrep.2017.02.012 28273461 PMC5357738

[B67] Ritzel R. M. Doran S. J. Glaser E. P. Meadows V. E. Faden A. I. Stoica B. A. (2019). Old age increases microglial senescence, exacerbates secondary neuroinflammation, and worsens neurological outcomes after acute traumatic brain injury in mice. *Neurobiol. Aging* 77 194–206. 10.1016/j.neurobiolaging.2019.02.010 30904769 PMC6486858

[B68] Russell A. L. Richardson M. R. Bauman B. M. Hernandez I. M. Saperstein S. Handa R. J. (2018). Differential responses of the HPA axis to mild blast traumatic brain injury in male and female mice. *Endocrinology* 159 2363–2375. 29701827 10.1210/en.2018-00203

[B69] Sah E. Krishnamurthy S. Ahmidouch M. Y. Gillispie G. J. Milligan C. Orr M. E. (2021). The cellular senescence stress response in post-mitotic brain cells: Cell survival at the expense of tissue degeneration. *Life* 11:229. 10.3390/life11030229 33799628 PMC7998276

[B70] Schwab N. Grenier K. Hazrati L.-N. (2019). DNA repair deficiency and senescence in concussed professional athletes involved in contact sports. *Acta Neuropathol. Commun.* 7:182. 10.1186/s40478-019-0822-3 31727161 PMC6857343

[B71] Schwab N. Ju Y. Hazrati L.-N. (2021). Early onset senescence and cognitive impairment in a murine model of repeated mTBI. *Acta Neuropathol. Commun.* 9:82. 10.1186/s40478-021-01190-x 33964983 PMC8106230

[B72] Simkin D. Hattori S. Ybarra N. Musial T. F. Buss E. W. Richter H. (2015). Aging-related hyperexcitability in CA3 pyramidal neurons is mediated by enhanced A-Type K+ channel function and expression. *J. Neurosci.* 35 13206–13218. 10.1523/JNEUROSCI.0193-15.2015 26400949 PMC4579378

[B73] Soto-Gamez A. Quax W. J. Demaria M. (2019). Regulation of survival networks in senescent cells: From mechanisms to interventions. *J. Mol. Biol.* 431 2629–2643. 10.1016/j.jmb.2019.05.036 31153901

[B74] Stojanovski S. Nazeri A. Lepage C. Ameis S. Voineskos A. N. Wheeler A. L. (2019b). Microstructural abnormalities in deep and superficial white matter in youths with mild traumatic brain injury. *Neuroimage Clin.* 24:102102. 10.1016/j.nicl.2019.102102 31795058 PMC6889799

[B75] Stojanovski S. Felsky D. Viviano J. D. Shahab S. Bangali R. Burton C. L. (2019a). Polygenic risk and neural substrates of attention-deficit/hyperactivity disorder symptoms in youths with a history of mild traumatic brain injury. *Biol. Psychiatry* 85 408–416. 10.1016/j.biopsych.2018.06.024 30119875 PMC6330150

[B76] Storer M. Mas A. Robert-Moreno A. Pecoraro M. Ortells M. C. Di Giacomo V. (2013). Senescence is a developmental mechanism that contributes to embryonic growth and patterning. *Cell* 155 1119–1130. 10.1016/j.cell.2013.10.041 24238961

[B77] Subramanian A. Tamayo P. Mootha V. K. Mukherjee S. Ebert B. L. Gillette M. A. (2005). Gene set enrichment analysis: A knowledge-based approach for interpreting genome-wide expression profiles. *Proc. Natl. Acad. Sci. U.S.A.* 102 15545–15550. 10.1073/pnas.0506580102 16199517 PMC1239896

[B78] Tominaga T. Shimada R. Okada Y. Kawamata T. Kibayashi K. (2019). Senescence-associated-β-galactosidase staining following traumatic brain injury in the mouse cerebrum. *PLoS One* 14:e0213673. 10.1371/journal.pone.0213673 30856215 PMC6411151

[B79] Torres-Querol C. Torres P. Vidal N. Portero-Otín M. Arque G. Purroy F. (2021). Acute ischemic stroke triggers a cellular senescence-associated secretory phenotype. *Sci. Rep.* 11:15752. 10.1038/s41598-021-95344-5 34344977 PMC8333348

[B80] van der Meer P. Ulrich A. M. Gonźalez-Scarano F. Lavi E. (2000). Immunohistochemical analysis of CCR2, CCR3, CCR5, and CXCR4 in the human brain: Potential mechanisms for HIV dementia. *Exp. Mol. Pathol.* 69 192–201. 10.1006/exmp.2000.2336 11115360

[B81] Vazquez-Villaseñor I. Garwood C. J. Heath P. R. Simpson J. E. Ince P. G. Wharton S. B. (2020). Expression of p16 and p21 in the frontal association cortex of ALS/MND brains suggests neuronal cell cycle dysregulation and astrocyte senescence in early stages of the disease. *Neuropathol. Appl. Neurobiol.* 46 171–185. 10.1111/nan.12559 31077599 PMC7217199

[B82] Velayudhan P. S. Schwab N. Hazrati L.-N. Wheeler A. L. (2021). Temporal patterns of microglial activation in white matter following experimental mild traumatic brain injury: A systematic literature review. *Acta Neuropathol. Commun.* 9:197. 10.1186/s40478-021-01297-1 34924026 PMC8684664

[B83] Wang Q. Duan L. Li X. Wang Y. Guo W. Guan F. (2022). Glucose metabolism, neural cell senescence and Alzheimer’s disease. *Int. J. Mol. Sci.* 23:4351. 10.3390/ijms23084351 35457168 PMC9030802

[B84] Weiland T. Lampe J. Essmann F. Venturelli S. Berger A. Bossow S. (2014). Enhanced killing of therapy-induced senescent tumor cells by oncolytic measles vaccine viruses. *Int. J. Cancer* 134 235–243. 10.1002/ijc.28350 23797800

[B85] Wilkinson H. N. Hardman M. J. (2020). Senescence in wound repair: Emerging strategies to target chronic healing wounds. *Front. Cell Dev. Biol.* 8:773. 10.3389/fcell.2020.00773 32850866 PMC7431694

[B86] Wilson W. H. O’Connor O. A. Czuczman M. S. LaCasce A. S. Gerecitano J. F. Leonard J. P. (2010). Navitoclax, a targeted high-affinity inhibitor of BCL-2, in lymphoid malignancies: A phase 1 dose-escalation study of safety, pharmacokinetics, pharmacodynamics, and antitumour activity. *Lancet Oncol.* 11 1149–1159. 10.1016/S1470-2045(10)70261-8 21094089 PMC3025495

[B87] Xu M. Pirtskhalava T. Farr J. N. Weigand B. M. Palmer A. K. Weivoda M. M. (2018). Senolytics improve physical function and increase lifespan in old age. *Nat. Med.* 24 1246–1256. 10.1038/s41591-018-0092-9 29988130 PMC6082705

[B88] Yang H. Wang H. Ren J. Chen Q. Chen Z. J. (2017). cGAS is essential for cellular senescence. *Proc. Natl. Acad. Sci. U.S.A.* 114 E4612–E4620. 10.1073/pnas.1705499114 28533362 PMC5468617

[B89] Yang J. Liu M. Hong D. Zeng M. Zhang X. (2021). The paradoxical role of cellular senescence in cancer. *Front. Cell Dev. Biol.* 9:722205. 10.3389/fcell.2021.722205 34458273 PMC8388842

[B90] Zeisel A. Hochgerner H. Lönnerberg P. Johnsson A. Memic F. van der Zwan J. (2018). Molecular architecture of the mouse nervous system. *Cell* 174 999–1014.e22. 10.1016/j.cell.2018.06.021 30096314 PMC6086934

[B91] Zhang J. Wang H. Sherbini O. Ling-Lin Pai E. Kang S.-U. Kwon J.-S. (2016). High-content genome-wide RNAi screen reveals CCR3 as a key mediator of neuronal cell death. *eNeuro* 3:ENEURO.0185-16.2016. 10.1523/ENEURO.0185-16.2016 27822494 PMC5075945

[B92] Zhang P. Kishimoto Y. Grammatikakis I. Gottimukkala K. Cutler R. G. Zhang S. (2019). Senolytic therapy alleviates Aβ-associated oligodendrocyte progenitor cell senescence and cognitive deficits in an Alzheimer’s disease model. *Nat. Neurosci.* 22 719–728. 10.1038/s41593-019-0372-9 30936558 PMC6605052

[B93] Zhu Y. Tchkonia T. Pirtskhalava T. Gower A. C. Ding H. Giorgadze N. (2015). The Achilles’ heel of senescent cells: From transcriptome to senolytic drugs. *Aging Cell* 14 644–658. 10.1111/acel.12344 25754370 PMC4531078

[B94] Zimmerman K. D. Espeland M. A. Langefeld C. D. (2021). A practical solution to pseudoreplication bias in single-cell studies. *Nat. Commun.* 12:738. 10.1038/s41467-021-21038-1 33531494 PMC7854630

[B95] Zumerle S. Alimonti A. (2020). In and out from senescence. *Nat. Cell Biol.* 22 753–754. 10.1038/s41556-020-0540-x 32591745

